# Epigenetic Control of IFN-γ Host Responses During Infection With *Toxoplasma gondii*

**DOI:** 10.3389/fimmu.2020.581241

**Published:** 2020-09-25

**Authors:** Roswitha Nast, Tenzin Choepak, Carsten G. K. Lüder

**Affiliations:** Institute for Medical Microbiology, University Medical Center Goettingen, Georg-August-University, Göttingen, Germany

**Keywords:** interferon-γ, gene expression, *Toxoplasma gondii*, immune evasion, epigenetics, chromatin, histone modification, DNA methylation

## Abstract

Host defense against the human pathogen *Toxoplasma gondii* depends on secretion of interferon (IFN)-γ and subsequent activation of monocytic cells to combat intracellular parasites. Previous studies have shown that *T. gondii* evades IFN-γ-mediated immunity by secreting the effector TgIST into the host cell where it binds to STAT1, strengthens its DNA binding activity and recruits the Mi-2/NuRD complex to STAT1-responsive promoters. Here we investigated the impact of the host chromatin environment on parasite interference with IFN-γ-induced gene expression. Luciferase reporters under control of primary and secondary IFN-γ response promoters were only inhibited by *T. gondii* when they were stably integrated into the host genome but not when expressed from a plasmid vector. Absence of CpG islands upstream and/or downstream of the transcriptional start site allowed more vigorous up-regulation by IFN-γ as compared to CpG-rich promoters. Remarkably, it also favored parasite interference with IFN-γ-induced gene expression indicating that nucleosome occupancy at IFN-γ-responsive promoters is important. Promoter DNA of IFN-γ-responsive genes remained largely non-methylated in *T. gondii*-infected cells, and inhibition of DNA methylation did not impact parasite interference with host responses. IFN-γ up-regulated histone marks H4ac, H3K9ac, and H3K4me3 but down-regulated H3S10p at primary and secondary response promoters. Infection with *T. gondii* abolished histone modification, whereas total nuclear activities of histone acetyl transferases and histone deacetylases were not altered. Taken together, our study reveals a critical impact of the host chromatin landscape at IFN-γ-activated promoters on their inhibition by *T. gondii* with a comprehensive blockade of histone modifications at parasite-inactivated promoters.

## Introduction

The intracellular parasite *Toxoplasma gondii* is a ubiquitous pathogen infecting birds and mammals including up to 30% of humans world-wide. While infections of immunocompetent hosts are commonly asymptomatic to benign, they can be severe to even life-threatening in immunocompromised hosts or after transmission to fetuses during pregnancy (1). *T. gondii* is also a significant cause of posterior uveitis after infection of immunocompetent adults (2), particularly in South America where hypervirulent strains of the parasite are common (3). In the U.S.A., toxoplasmosis has recently been recognized as a leading food-borne infectious disease based on annual costs and loss of quality-adjusted life years (4).

*T. gondii* actively invades various host cell types including monocytic cells and extensively modifies host signaling pathways and dampens anti-parasitic effector mechanisms [recently reviewed in (5, 6)]. This facilitates replication of the tachyzoite stage within a membrane-bound parasitophorous vacuole (PV). It also allows dissemination to distant organs including brain and muscle tissues (7, 8) where the parasite transforms into a latent stage, i.e., the bradyzoite. Bradyzoites persist for months to years, and they are critical for transmission to new hosts. Host cell modulation by *T. gondii* requires injection of secreted effector proteins of the rhoptry family (ROPs) into the host cell during invasion (9, 10), or translocation of dense granule effector proteins (GRAs) across the PV membrane (11–13) in a MYR1- and/or ASP5-dependent process (14–16).

One of the critical host pathways that is severely impaired in infected cells is their responsiveness to IFN-γ (17–20). IFN-γ regulates expression of >1,000 genes involved in cell-autonomous immune defense, regulation of immune responses and immune-unrelated processes (21). After binding to its receptor, it activates signal transducer and activator of transcription (STAT)-1, which enters the nucleus and binds to gamma-activated site (GAS) motifs in the promoters of IFN-γ responsive genes (22). IFN-γ responses are induced in multiple waves with expression of primary response genes being initiated by active STAT1 alone, while transcription of secondary and tertiary response genes requires additional transcription factors expressed during the first round of expression. Both IFN-γ (23) and STAT1 (24, 25) are essential for efficient control of *T. gondii* infections. Recently, the GRA protein TgIST was shown translocating into the host cell in an ASP5-dependent manner, entering the host cell nucleus and repressing IFN-γ-regulated gene transcription (26, 27). TgIST binds to activated STAT1 complexes (20, 26, 28) and recruits the Mi-2/NuRD chromatin repressor complex to STAT1-responsive promoters (26, 27). This is associated with impaired chromatin remodeling at distinct IFN-γ-responsive promoters (20, 26), sequestration of STAT1 at GAS and non-GAS promoters (27–29) and impaired nuclear export and recycling of STAT1 (28, 29). How exactly TgIST inhibits IFN-γ-regulated gene transcription is however yet unknown. Importantly, parasites deficient in TgIST are unable to counteract IFN-γ responses of their host cells *in vitro*, and they are avirulent *in vivo* (26, 27).

Transcription of genes including those of an inflammatory response requires a permissive three-dimensional chromatin structure that allows binding of transcription factors, chromatin modifiers and the transcriptional machinery at respective promoters (30). Posttranslational modifications (PTMs) of residues particularly within histones H3 and H4 tails are critical in regulating this process. Acetylation and phosphorylation neutralize or negatively charge histone domains thereby decreasing their interaction with DNA (31). Furthermore, methylation and to a lower extent also acetylation and phosphorylation of histones can enable or prevent recruitment of diverse chromatin-modifying enzymes. Mono-, di- or tri-methylation, and cross-talk between different histone PTMs further contribute to regulation of transcription. Methylation of DNA at the cytosine of CpG dinucleotides is another epigenetic mark that regulates gene transcription, with non-methylated CpGs allowing and methylated CpGs restricting promoter activation, respectively (32). Whereas vertebrate genomes are generally CpG-poor due to the mutagenic potential of methyl-cytosine, 60–70% of promoters contain an elevated number of CpGs referred to as CpG islands (CGI) (33, 34), though mostly non-methylated in normal cells (35).

The Mi-2/NuRD complex that is recruited to STAT1-responsive promoters in *T. gondii*-infected cells in a TgIST-dependent manner is a multi-subunit complex comprising histone deacetylases (HDAC) 1 and 2 and methyl-CpG-binding domain-containing protein (MBD) 2 and 3, among other components (36). It may thus silence gene expression by histone deacetylation (36) and/or by increasing DNA methylation (36–38).

To better understand how IFN-γ-regulated gene expression is inhibited in *T. gondii*-infected cells, we herein performed an in-depth analysis of the host chromatin at promoters of representative primary and secondary response genes. We for the first time directly identify the critical impact native chromatin has on the evasion of IFN-γ responses by *T. gondii*. The parasite's ability to abrogate IFN-γ-induced gene expression is favored at promoters lacking CGIs, and consistently, it does not rely on DNA methylation. In contrast, it coincides with broadly counteracting IFN-γ-regulated histone modifications at both primary and secondary response genes.

## Results

### Inhibition of IFN-γ-Regulated Gene Expression by *T. gondii* Requires Native Chromatin Environment

**Figure 1:**
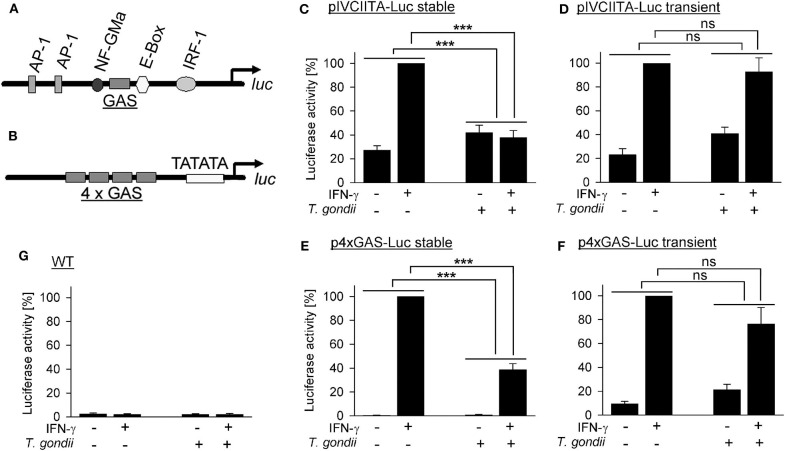
Inhibition of primary and secondary IFN-γ responses of monocytic cells by *T. gondii* requires a native chromatin environment. RAW264.7 monocytes/macrophages were stably **(C,E)** or transiently **(D,F)** transfected with luciferase reporters under control of a truncated version of the *cIIta* promoter IV **(A)** or a minimal 4 × GAS promoter **(B)**. **(C–G)** Cells were infected with *T. gondii* for 24 h or left non-infected and stimulated or not with IFN-γ at 3 h p.i. as indicated. Luciferase activities in lysates from equal amounts of cells were determined by luminescence measurements; lysates from non-transfected wild type cells were measured in parallel **(G)**. Bars represent mean percent luciferase activities ± S.E.M. (six biological replicates) as compared to non-infected cells after stimulation with IFN-γ (100%); differences between groups were evaluated by ANOVA (***p < 0.001; ns, not significant).

The *T. gondii* effector TgIST, after translocation into the host cell, recruits Mi-2/NuRD to STAT1-responsive promoters (26, 27) and represses IFN-γ responses of its host cell (17, 19, 20, 26, 27). It also increases binding of STAT1 to naked DNA oligonucleotides *in vitro* and to native genomic DNA in infected cells, thereby diminishing recycling and reactivation of STAT1 (28, 29). Here, we have directly determined whether a native chromatin environment is required for *T. gondii* to inhibit STAT1-dependent gene transcription in infected monocytic cells. RAW264.7 cells were stably transfected with *luc* under control of a 5'-truncated version of the endogenous *cIIta* promoter IV (pIV; Figure 1A), representing a secondary response gene. Non-infected cells up-regulated luciferase activity ~4-fold upon activation with IFN-γ (Figure 1C). Infection with *T. gondii* rendered cells unable to respond to IFN-γ, as expected from our previous results (39). Remarkably, when cells were transiently transfected with the *cIIta* pIV-driven reporter, *T. gondii* infection did not abrogate IFN-γ responsiveness (Figure 1D), indicating that a native chromatin environment is required for the parasite to inhibit IFN-γ-induced activation of this promoter. Gene expression may be differently regulated by the chromatin environments of primary and secondary response genes (40). Therefore, we next stably transfected RAW264.7 cells with *luc* downstream of a minimal 4xGAS promoter which is regulated by activated STAT1 only and thus represents a primary response gene (Figure 1B). These cells up-regulated luciferase activity in response to IFN-γ ~200-fold when being non-infected, but only ~40-fold after infection with *T. gondii* (Figure 1E; *p* < 0.001; ANOVA). The higher regulation of luciferase as compared to pIV*cIIta*-*luc* cells may be due to synergistic binding of STAT1 tetramers to adjacent GAS motifs (41, 42). More importantly however, after transient transfection with p4xGAS-*luc, T. gondii* only slightly inhibited IFN-γ-induced activity (Figure 1F; *p* > 0.05). Wild-type cells did not show any significant luciferase activity (Figure 1G). In unstimulated *luc*-transfected cells, infection with *T. gondii* slightly increased reporter activity (Figures 1C–F), consistent with increased binding of STAT1 to DNA in infected cells, and possibly depending on ROP16 as reported for type I parasites (20, 28, 29). Together, these results directly establish a critical impact of native host chromatin on the ability of *T. gondii* to counteract IFN-γ-dependent activation of both primary and secondary response genes.

### Lack of CpG Islands Favors *T. gondii* Interference With IFN-γ-Induced Gene Expression

**Figure 2:**
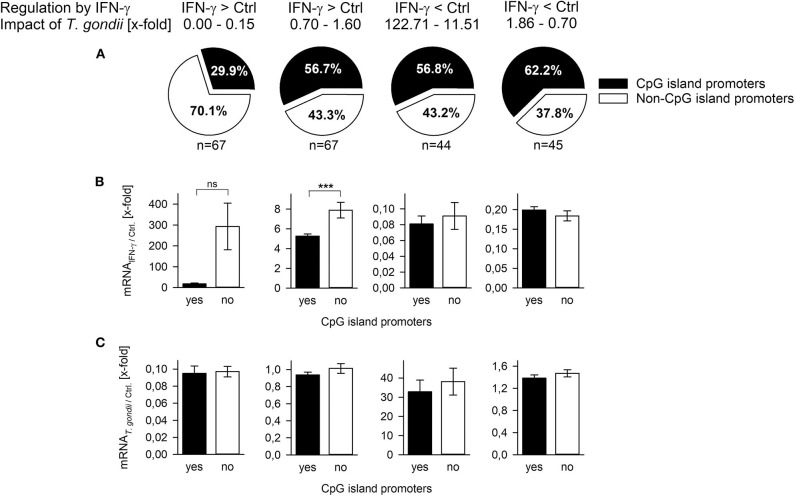
*T. gondii* preferentially inhibits expression of IFN-γ-induced genes regulated by non-CpG island promoters. Genes (*n* = 223) were selected from a previous microarray analysis (20), and they were grouped according to their regulation by IFN-γ (up- or down-regulated) and the x-fold regulation by concomitant infection with *T. gondii*. **(A)** They were *in silico* analyzed for presence or absence of CpG islands within nucleotides −200 to +200 relative to the transcriptional start site. Fold mRNA regulation induced by IFN-γ in non-infected cells **(B)** or by *T. gondii* compared to non-infected controls **(C)** were calculated for genes with or without CpG island promoters. Bars represent means ± S.E.M.; differences between groups were evaluated by Student's *t*-test (***p < 0.001; ns, not significant).

CpG islands (CGIs) hinder DNA bending around histone octamers, and CGI promoters are thus generally nucleosome-depleted and readily accessible to transcription factors and the basal transcriptional machinery [reviewed in (43)]. CGI promoters of inflammatory response genes differ from non-CGI promoters by their activation independently of nucleosome remodeling by SWI/SNF (40) and by their lower fold induction after stimulation (44). Here, we made use of a previous genome-wide microarray analysis of *T. gondii*-infected and control mouse macrophages during stimulation with IFN-γ (20) to decipher the role of CGIs in the ability of *T. gondii* to counteract IFN-γ responses. We selected 67 IFN-γ-induced genes each, expression of which was either strongly repressed by the parasite (0.00–0.15-fold) or not repressed (0.7–1.6-fold; Figure 2A). Although mechanisms regulating repression of genes by IFN-γ are only known for ~15% of them (45), we also selected ~45 IFN-γ-repressed genes each being either strongly induced after infection (11.51–122.71-fold) or not (0.7–1.86-fold). Only ~30% of those IFN-γ-induced genes that were strongly inhibited by parasite infection contained CGIs in their promoters (Figure 2A, Supplementary Table 1), thus significantly deviating from the overall ~60% of CGI promoters in the mouse genome (33). In contrast, 56.7–62.2% of the IFN-γ-induced genes not being repressed by *T. gondii* and of the IFN-γ-repressed genes irrespective of being induced by *T. gondii* or not, were characterized by CGI promoters, being in line with the average percentage in the mouse genome (Figure 2A, Supplementary Table 1). IFN-γ-induced genes with non-CGI promoters were generally more strongly expressed than those with CGI promoters, although this differed statistically only for those not being counteracted by the parasite (*p* < 0.001, Student's *t*-test; Figure 2B), consistent with previous findings for lipopolysaccharide (LPS)-induced genes (44). Furthermore, overall fold up-regulation by IFN-γ irrespective of CpG occupancy of their promoters differed significantly between genes being either repressed by *T. gondii* (210.99 ± 79.41) or not (6.39 ± 0.4; *p* = 0.011, Students *t*-test; not shown). In contrast, the impact *T. gondii* had on the gene regulation by IFN-γ did not differ between CGI promoters and non-CGI promoters (Figure 2C). Together, results indicate that among the IFN-γ-inducible genes, *T. gondii* primarily inhibits those lacking CGIs in their promoters and thus favoring strong induction by IFN-γ.

### DNA Methylation Does Not Impact *T. gondii*-Mediated Host Cell Unresponsiveness to IFN-γ

**Figure 3:**
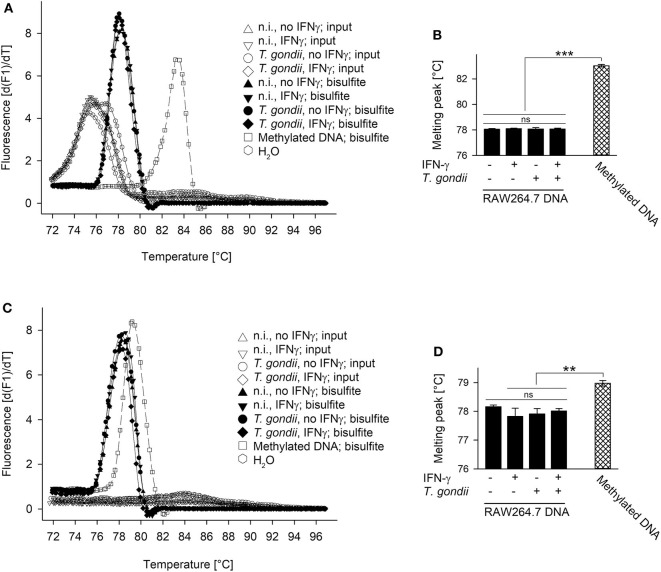
Promoters of *irf1* and *cIIta* (pIV) in monocytic cells remain non-methylated during infection with *T. gondii* and/or stimulation with IFN-γ. RAW264.7 cells were infected or not with *T. gondi* and were stimulated or not with IFN-γ at 3 h p.i. DNA was isolated from the cells 24 h after infection and was subjected to bisulfite conversion; universally methylated DNA was treated in parallel and used as standard. Promoter regions of *irf1* **(A,B)** and *cIIta* **(C,D)** were qPCR-amplified from input sample DNA and from bisulfite-treated sample or standard DNA using methylation-independent primer pairs. Amplicons were subjected to methylation-sensitive melting curve analysis; **(A,C)** melting peaks were visualized by plotting the first negative derivative of fluorescence with respect to temperature vs. temperature. **(B,D)** Bars represent mean melting peaks ± S.E.M. (*n* = 5) of amplicons from bisulfite-treated sample and standard DNA as calculated by the LightCycler software; significant differences were identified by ANOVA (**p < 0.01; ***p < 0.001; ns, not significant).

The Mi-2/NuRD repressor binds methylated DNA via methyl-binding domain protein (MDB) 2 (36, 46). Furthermore, the complex recruited by TgIST to STAT1-dependent promoters in *T. gondii*-infected cells contains the highly related MDB3 (26, 27). Finally, the IFN-γ-responsive pIV of *cIIta* is silenced in fetal trophoblast cells (47, 48) and in cancer cells (49) by DNA methylation. We therefore tested the hypothesis of unresponsiveness of *T. gondii*-infected cells to IFN-γ being linked to increased methylation of cytosines at STAT1-responsive promoters. Genomic DNA from *T. gondii*-infected and non-infected RAW264.7 cells stimulated or not with IFN-γ was bisulfite-treated, and then analyzed using methylation-sensitive melting curve analysis (50). The PCR amplicon of an *irf1* promoter region yielded a melting peak that specifically differed from non-bisulfite-treated input DNA, consistent with conversion of non-methylated cytosines to uracil (Figure 3A). More importantly, melting peaks did not differ between cells being parasite-infected or not and/or stimulated with IFN-γ or not indicating highly similar DNA methylation patterns (Figures 3A,B). Universally methylated control mouse DNA, after bisulfite conversion, yielded a melting peak at significantly higher temperature than DNA from RAW264.7 samples (*p* < 0.001, ANOVA; Figures 3A,B). This is consistent with methyl-cytosines remaining unchanged during bisulfite treatment, and the notion that CpGs within the *irf1* promoter are largely non-methylated in RAW264.7 cells before and after infection with *T. gondii*. Similarly, melting peaks of amplicons from pIV of *cIIta* did not differ between cells infected or non-infected and/or IFN-γ-treated or untreated. Their peak temperatures except that from unstimulated, non-infected cells were however significantly lower than that from methylated control DNA (Figures 3C,D) indicating that the corresponding promoters are also largely non-methylated.

**Figure 4:**
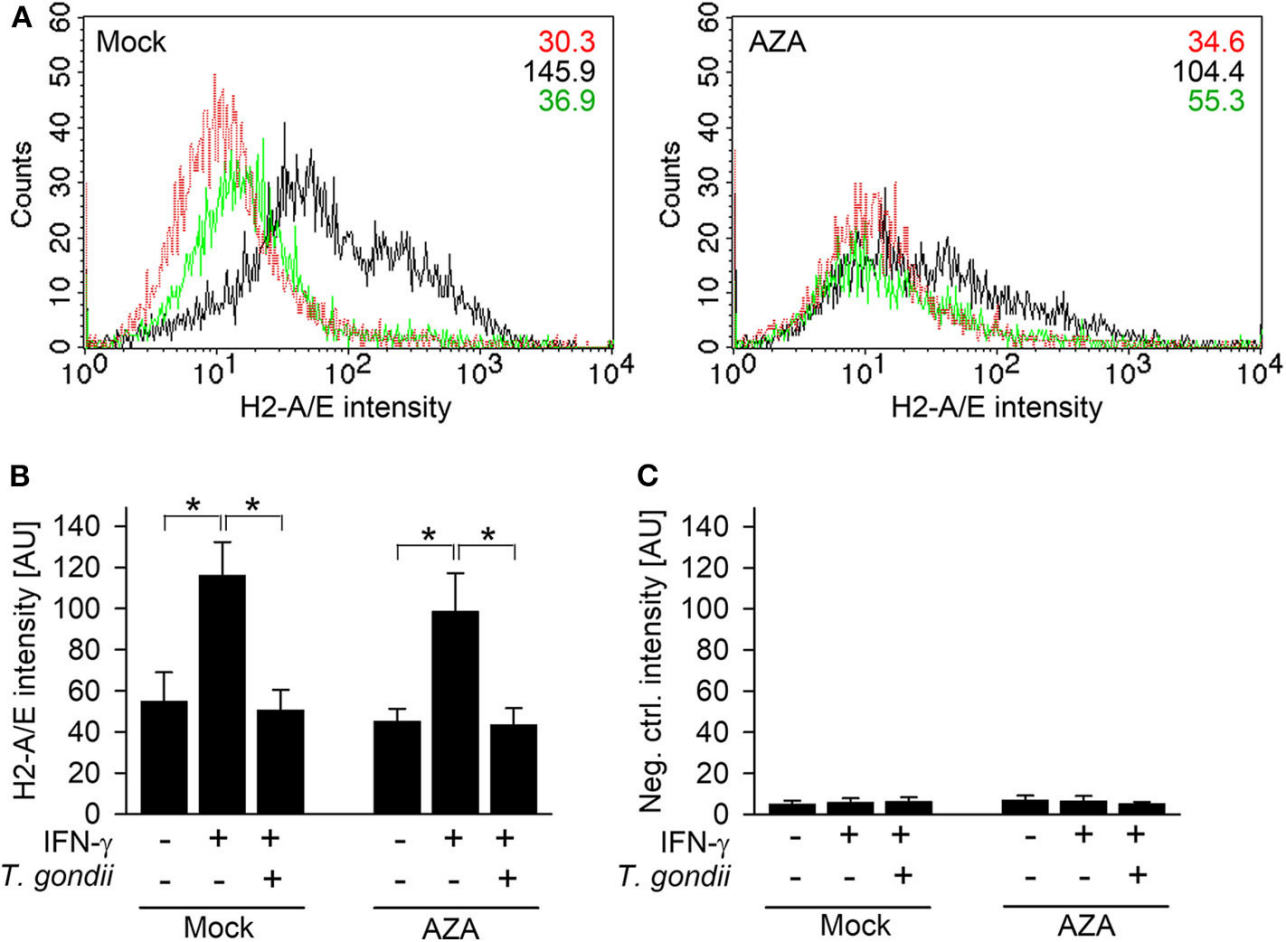
*T. gondii* blocks IFN-γ-induced MHC class II expression irrespective of inhibition of DNA methyltransferases. RAW264.7 cells were treated with 5-aza-2-deoxycytidine (AZA) for 7 days, before being infected with *T. gondii* and/or activated with IFN-γ. Surface expression of MHC class II was determined at ~42 h p.i. by flow cytometry, after fluorescence staining of cells using H2-A/E-specific antibodies **(A,B)** or isotype control antibodies **(C)**. **(A)** Representative histograms of mock- and AZA-treated cells being left non-activated, non-infected (red), IFN-γ-activated (black), or *T. gondii*-infected and IFN-γ-activated (green); figures within the plots indicate mean fluorescence intensities of the respective cell population. **(B,C)** Bars represent mean fluorescence intensities ± S.E.M. from five biological replicates; significant differences between groups were identified by ANOVA (*p < 0.05).

The impact of DNA methylation on IFN-γ-unresponsiveness of *T. gondii*-infected cells was further validated by quantitating H2-A/E molecules on RAW264.7 cells treated or not treated for 7 days with 5-aza-2-deoxycytidine (AZA), i.e., an irreversible inhibitor of DNA methyltransferases. H2-A/E are *bona fide* IFN-γ-regulated molecules, which we have routinely measured in the past by FACS to confirm inhibition of their IFN-γ-regulated up-regulation in macrophages infected with *T. gondii* (17, 20). Furthermore, H2-A/E expression depends on both primary (IRF1) and secondary (CIITA) IFN-γ-regulated transcription factors, and this increases likelihood to detect any effect of AZA on IFN-γ-regulated gene expression in infected cells. AZA-treated cells significantly up-regulated H2-A/E molecules in response to IFN-γ similar to mock-treated cells (*p* < 0.05, ANOVA; Figures 4A,B), though to slightly lower extent. Up-regulation of H2-A/E in response to IFN-γ was abolished by previous infection with *T. gondii*, both in AZA- and mock-treated cells (*p* < 0.05; Figures 4A,B). Staining of cells with an isotype control antibody, irrespective of being treated with AZA or not, yielded background fluorescence only (Figure 4C). Collectively, these results establish that DNA methylation does not mediate unresponsiveness of *T. gondii*-infected monocytes/macrophages to IFN-γ.

### Regulation of Histone Marks During IFN-γ-Responses Are Broadly Inhibited by *T. gondii*

**Figure 5:**
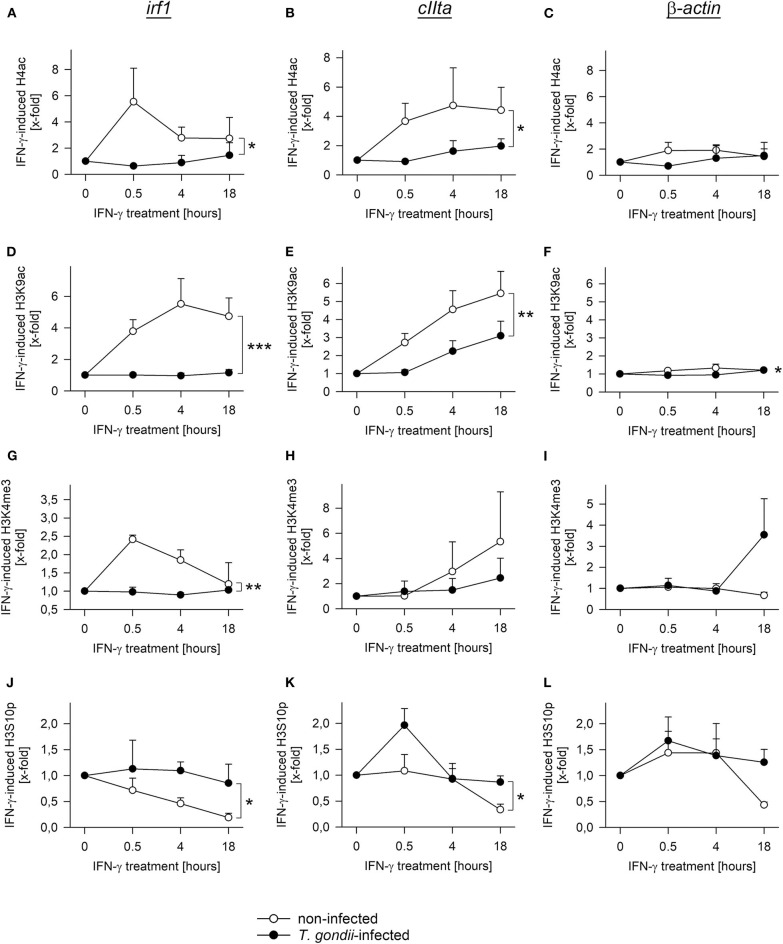
*T. gondii* largely abolishes regulation of diverse histone modifications during IFN-γ stimulation of RAW264.7 cells. Cells were parasite-infected for 24 h or were left non-infected. During the final 0.5–18 h, they were stimulated with IFN-γ or were left unstimulated (0 h). After cross-linking DNA-protein complexes, cell lysates were subjected to ChIP using antibodies binding to pan-acetyl-H4 **(A–C)**, acetylK9-H3 **(D–F)**, tri-methylK4-H3 **(G–I)** or phosphoS10-H3 **(J–L)**. After isolation of DNA from chromatin immunoprecipitates or from input chromatin, fragments of promoters of *irf1* **(A,D,G,J)**, *cIIta* (pIV; **B,E,H,K**) and β*-actin* **(C,F,I,L)** were amplified by qPCR. Data indicates means ± S.E.M. (two to six biological replicates) of cytokine-induced regulation of histone modifications in *T. gondii*-infected (closed symbols) and non-infected (open symbols) cells normalized to input DNA; differences between experimental groups were identified by ANOVA (*p < 0.05; **p < 0.01; ***p < 0.001).

During activation by IFN-γ, acetylation of histones at promoters of secondary response genes is inhibited by prior infection with *T. gondii* (20, 26). We therefore wondered how other histone marks may change in infected and non-infected cells during an IFN-γ response, how they are regulated over time, and whether they differ between primary and secondary response genes. Chromatin immunoprecipitation (ChIP) revealed pan-acetylation of histone H4 (H4ac) and tri-methylation of H3 at lysine 4 (H3K4me3) at the *irf1* promoter within 30 min of IFN-γ activation in non-infected RAW264.7 cells and a decline until 18 h of stimulation (Figures 5A,G). Prior infection of cells with *T. gondii* completely abolished up-regulation of H4ac and H3K4me3 (*p* < 0.05 for H4ac, *p* < 0.01 for H3K4me3, ANOVA). Acetylation of H3K9 showed a slower increase with a peak at 4 h of stimulation in non-infected cells, but was also abrogated by prior infection (Figure 5D; *p* < 0.001). H3K9ac was similarly regulated at the promoters of the primary response genes *irf8* and *stat1*, and it was also significantly inhibited by *T. gondii* (Supplementary Figures 1A,B; *p* < 0.05). Remarkably, phosphorylation of H3 at serine 10 (H3S10p) steadily decreased in non-infected cells in response to IFN-γ (Figure 5J), indicating that its presence marks non-activated STAT1-responsive promoters. Importantly, the decrease of H3S10p was also largely inhibited by *T. gondii* (*p* < 0.05). Thus, histone modification as observed at the *irf1* promoter in response to IFN-γ is broadly and almost completely prevented by *T. gondii* infection. H4ac, H3K9ac, and H3K4me3 also increased and H3S10p decreased in response to IFN-γ at the pIV*cIIta* promoter, though with delayed kinetics compared to the *irf1* promoter as expected (Figures 5B,E,H,K). In contrast, cells infected with *T. gondii* were largely unable to regulate these modifications after IFN-γ activation. The induction of H3K9ac was also significantly reduced by *T. gondii* at the promoter of *gbp2*, i.e., another secondary response gene (Supplementary Figure 1C; *p* < 0.01). At the constitutive promoter of β*-actin*, H4ac, H3K9ac, H3K4me3, and H3S10p did not considerably differ between parasite-infected and non-infected cells during activation with IFN-γ (Figures 5C,F,I,L). Collectively, these results establish a severe defect of *T. gondii*-infected monocytes/macrophages to regulate different histone marks including acetylation, methylation and phosphorylation at both primary and secondary IFN-γ response genes.

**Figure 6:**
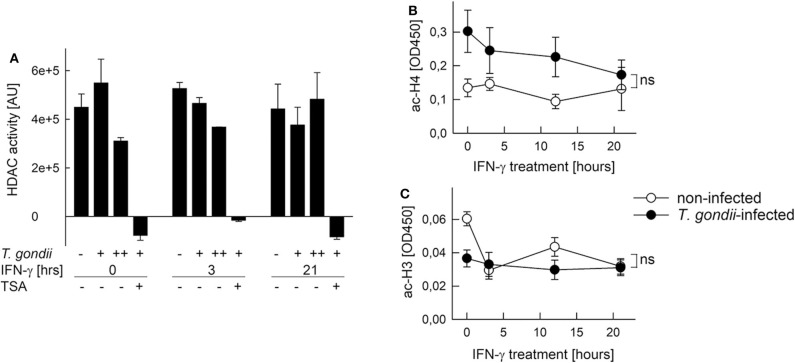
Histone acetylation at IFN-γ-responsive promoters is inhibited by *T. gondii* without generally impacting HDAC and HAT activities in nuclei of infected cells. RAW264.7 monocytes/macrophages were infected with *T. gondii* at parasite-to-host cell ratios of 3:1 (**A**: +) or 6:1 (**A**: ++; **B,C**) for 24 h or were left non-infected, and they were stimulated with IFN-γ during the final 3–21 h or left non-stimulated (0 h). **(A)** HDAC activity was determined in nuclear extracts by measuring deacetylation of a synthetic substrate fluorometrically; some test reactions were performed in presence of the HDAC inhibitor trichostatin A (TSA). Bars represent means ± S.E.M. from two biological replicates. **(B,C)** Acetylation of synthetic histone H4 **(B)** and H3 **(C)** peptides by nuclear extracts was determined by a colorimetric ELISA; data represent means ± S.E.M. (five biological replicates; ns, not significant, ANOVA).

Histone acetylation is critical to activate gene expression (51). Furthermore, Mi-2/NuRD recruited by *T. gondii* to STAT1-responsive promoters contains histone deacetylases (HDAC) 1 and 2 (26, 27), and HDAC inhibitors partially rescue *T. gondii*-infected monocytic cells to respond to IFN-γ (20, 29). Therefore, we next tested the possibility that HDAC or histone acetyl transferase (HAT) activities are altered after infection. HDAC activity in nuclear extracts of RAW264.7 cells was not significantly altered after infection with increasing amounts of *T. gondii* as compared to non-infected controls (Figure 6A). It did also not change following stimulation with IFN-γ for 3 or 21 h, irrespective of being infected or not. The HDAC inhibitor trichostatin A (TSA) completely abolished HDAC activity, confirming specificity of the assay (Figure 6A). Further, NAD^+^ within the reaction buffer did not impact measurements, indicating that sirtuins do not contribute to the overall nuclear HDAC activity in RAW264.7 cells (data not shown). Acetylation of a histone H4 peptide by HATs was higher, though statistically not significantly, in nuclear extracts from *T. gondii*-infected RAW264.7 cells throughout stimulation with IFN-γ for 0–21 h, compared to non-infected controls (Figure 6B). In addition, HAT activity toward a histone H3 peptide was impaired in nuclear extracts from infected but non-activated cells (0 h), but did not differ during stimulation with IFN-γ (Figure 6C). Results thus establish that overall nucleoplasmic HDAC and HAT activities do not contribute to IFN-γ unresponsiveness of monocytes/macrophages during *T. gondii* infection.

## Discussion

Infection by *T. gondii* renders host cells including immune cells largely unresponsive to IFN-γ (17–20), and this is critical for parasite survival and virulence during acute infection (26, 27). We now demonstrate that *T. gondii* inhibits IFN-γ-dependent gene expression (i) only at promoters with a native chromatin environment, and (ii) predominantly at those lacking CpG islands. Consistent with these findings, (iii) were histone modifications at promoters of primary and secondary response genes broadly ablated by *T. gondii*, whereas (iv) DNA methylation was unaffected during infection. These results further our understanding of how *T. gondii* manipulates cytokine responsiveness of monocytes/macrophages via interference with the chromatin environment at a subset of STAT1-responsive promoters. They also unveil new insights into the general regulation of IFN-γ-mediated gene expression in mouse monocytic cells.

*T. gondii* infection increases binding of activated STAT1 to host chromatin (28, 29) and to IFN-γ-responsive native promoters (26, 27, 29), and it impairs histone modifications at promoters of secondary response genes (20, 26). *T. gondii* however also potentiates binding of aberrant STAT1 complexes to naked DNA (20, 26, 28, 52) raising questions about the role of the host chromatin for repression of IFN-γ-mediated gene expression. Using luciferase reporters, we here provide direct experimental evidence that native chromatin is indispensable for *T. gondii* to inhibit IFN-γ-regulated gene expression. Only after stable integration of the transgene into the host cell genome but not when being expressed from a plasmid vector, was the reporter repressed. Further, both primary and secondary response promoters were silenced in a host chromatin-dependent manner. It is unlikely that high copy numbers of the transgene within transiently transfected cells and therefore exhaustion of *T. gondii* to inhibit *luc* expression account for this finding, since absolute luciferase activities were not consistently higher in these cells as compared to stable transfectants (data not shown). Also, only a small proportion of plasmid DNA reaches the nucleus and can get expressed (53). Our results instead suggest that the Mi-2/NuRD complex can only be recruited by TgIST to GAS promoters (26, 27) within a native chromatin context and/or that a repressive chromatin environment is crucial for inhibition of IFN-γ-triggered gene expression. The sole increased and sustained binding of STAT1 complexes from *T. gondii*-infected cells to naked DNA as described by us and others *in vitro* (20, 26, 28, 52) appears however to not suffice for repression of gene expression, as we expect such altered binding also occurring at GAS promoters within plasmid DNA.

**Figure 7:**
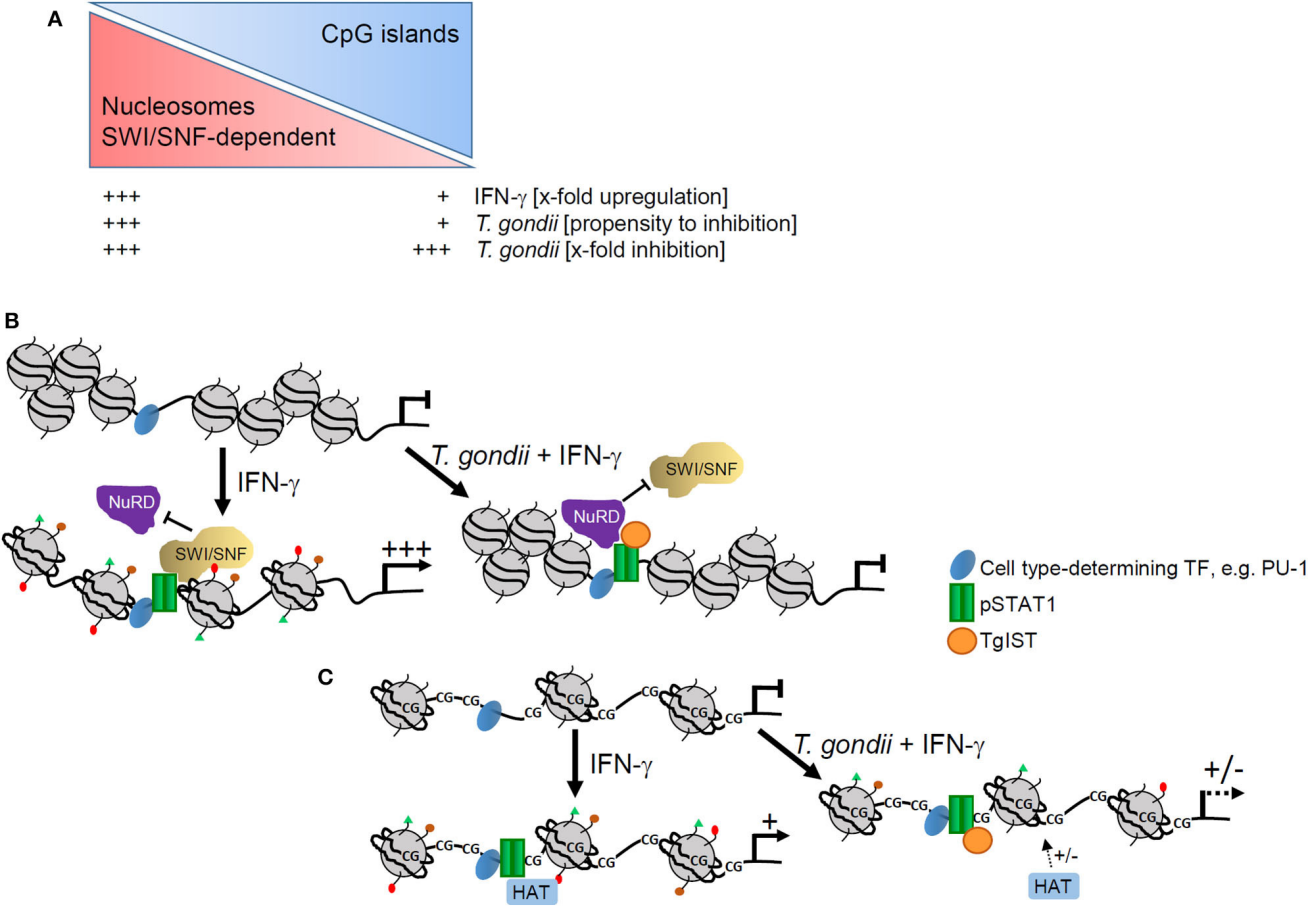
Model of epigenetic control of IFN-γ-induced gene expression by *T. gondii*. **(A)** Presence or absence of CpG islands impact magnitude of promoter activation by IFN-γ and propensity of *T. gondii* to counteract such activation, but it does not generally impact relative levels of parasite-mediated inhibition. **(B)** CpG-poor promoters are generally densely packed within nucleosomes and their activation by IFN-γ depends on extensive remodeling by SWI/SNF complexes. In *T. gondii* infected cells, TgIST favors binding of NuRD that is co-recruited together with SWI/SNF and represses promoter activation including nucleosome remodeling and histone modifications. **(C)** CpG islands within promoters instead favor an open chromatin that does not rely on extensive nucleosome remodeling. Activation of such promoters by IFN-γ requires distinct histone modifications, e.g., acetylation by histone acetyl transferases (HATs). Binding of TgIST to STAT1 in infected cells can abolish histone modifications with limited propensity.

We also provide strong evidence that *T. gondii* preferentially ablates activation of those promoters which are devoid of CpG islands (Figure 7A). In agreement with the ~40% CpG-poor promoters of the mouse genome (33), 38–43% of promoters whose IFN-γ-regulated activities were not significantly counteracted by *T. gondii* (20), were CpG-poor. In sharp contrast however, of the IFN-γ-induced promoters whose activation was strongly inhibited by the parasite (20), 70% were CpG-poor. This indicates that non-CpG island promoters structurally and/or mechanistically favor interference of *T. gondii* with their IFN-γ-induced activation. Absence of CpG islands promotes assembly of promoter DNA within nucleosomes (43), and imposes a requirement for SWI/SNF-dependent nucleosome remodeling for their activation, at least in response to NF-κB (40, 44). The promoter IV of the IFN-γ secondary response gene *cIIta* is also devoid of CpG islands (see Supplementary Table 1), and its activation depends on the SWI/SNF core subunit BRG-1 (54). We have previously confirmed significant recruitment of BRG-1 to DNA encompassing STAT1 consensus sequences including the *cIIta* pIV in non-infected, but not in *T. gondii*-infected macrophages in response to IFN-γ (20). In LPS-activated macrophages, the Mi-2/NuRD complex selectively antagonizes SWI/SNF-dependent activation of secondary and delayed primary response genes (55). In contrast, SWI/SNF-independent early primary response genes are not or only slightly repressed by Mi-2/NuRD (55). Along that line may TgIST predominantly recruit the Mi-2/NuRD complex in IFN-γ-activated macrophages to SWI/SNF-dependent, i.e., to CpG-poor promoters (Figure 7B). We thus propose a model in which binding of TgIST to STAT1 (26, 27) does not suffice to recruit Mi-2/NuRD to GAS promoters and repress their activation, but additionally requires a chromatin environment that is often CpG-poor, and dependent on SWI/SNF-mediated nucleosome remodeling for IFN-γ-induced activation. In contrast, CpG islands in promoters correlate with H3K4me3 and constitutive association with RNA polymerase II, that are features of active chromatin prior to stimulation (40, 56) and allows rapid gene expression after LPS stimulation (57). The majority of these promoters are not significantly regulated by Mi-2/NuRD (55). Likewise, independence of Mi-2/NuRD may explain why TgIST does not globally repress CpG-rich promoters in response to IFN-γ (Figure 7C). It is important to note that presence of CpG islands within IFN-inducible promoters does not *per se* preclude repression by *T. gondii* (see Supplementary Table 1). Whether distinct chromatin features enable parasite-mediated Mi-2/NuRD recruitment to these promoters despite presence of CpG islands, or whether gene expression is repressed by different means (28) is unknown.

In response to LPS, SWI/SNF-dependent, i.e., CpG-poor promoters in macrophages are more tightly regulated and show a higher dynamic range of regulation (44). Data presented herein now extends this conclusion to promoters that are activated in response to IFN-γ (see Figure 2B, left panels; Figure 7A). It does however not apply to promoters which are repressed upon IFN-γ treatment. In human macrophages, ~15% of repressed genes are downregulated by disassembly of enhancer regions (45), i.e., distal control elements that regulate gene expression in a tissue-specific manner. Suppression of the gene-activating histone mark H3K27ac at enhancers and promoters in response to IFN-γ might additionally regulate gene repression (45, 58). SWI/SNF and/or Mi-2/NuRD have not yet been related to gene repression by IFN-γ, and this may explain that presence or absence of CpG islands in promoters of these genes does not affect the magnitude of regulation (see Figure 2B, right panels). However, gene repression in response to IFN-γ clearly requires further clarification. It will also be of major interest to uncover how *T. gondii* counteracts IFN-γ-regulated gene repression mechanistically (20).

Consistent with a major impact of the chromatin environment on *T. gondii*-mediated inhibition of IFN-γ-induced gene expression are histone modifications broadly inhibited in infected monocytes/macrophages, whereas DNA methylation (47–49) does not have an impact. H4Kac including K5ac, K8ac and K91ac, H3K9ac and H3K4me3 are part of an acetylation/methylation histone code that correlates with gene activation in human T cells (59). Along that line, IFN-γ-induced increase of H4Kac, H3K9ac, and H3K4me3 were all inhibited at primary and secondary response gene promoters after parasite infection. Inhibition did not generally differ between promoters containing or not containing CpG islands, since it similarly occurred at the CpG-rich *irf1* and *stat1* promoters and the CpG-poor *cIIta* pIV and *gbp2* promoters (see Supplementary Table 1), although it is difficult to draw a general conclusion from this small number of promoters. Therefore, ChIP-seq analyses may in the future provide a genome-wide view on that issue. Phosphorylation of H3S10 decreased in non-infected, but not in *T. gondii*-infected cells in response to IFN-γ. This is remarkable, since H3S10p is associated with active gene expression in response to mitogens, stress signals and TLR ligands (60, 61). Our data now suggests, that in response to IFN-γ, H3S10p rather represents a repressive histone mark, at least at the *irf1* and *cIIta* pIV promoters. After stimulation of non-infected cells with IFN-γ, H4Kac and H3K4me3 appeared earlier at the *irf1* promoter than H3K9ac and loss of H3S10p, suggesting apical roles in activating promoters of primary response genes. H4Kac and H3K4me3 also peaked earlier at the *irf1* promoter than at the *cIIta* pIV, consistent with the different kinetics of promoter activation (48). Finally, *T. gondii* generally inhibited histone modifications more vigorously at primary response gene promoters than at secondary response promoters. The reason for the latter observation is unknown. Our data nevertheless clearly show a profound and broad inhibition of IFN-γ-triggered histone modifications by *T. gondii* thus confirming and extending previous findings (20, 26). Of note, histone modifications were selectively counteracted by *T. gondii* at IFN-γ-regulated promoters, indicating that *cis* control elements, most presumably GAS sites, are critical. In agreement, total cellular H4Kac level are not altered by parasite infection (Brand and Lüder, unpublished), nor are the nuclear activities of HATs and HDACs.

Together, we provide novel insights into epigenetic control of monocyte/macrophage gene expression in response to IFN-γ, and we identify a critical impact of the epigenomes at IFN-γ-responsive promoters on their inhibition by an intracellular parasite. Our results raise important future research directions on how TgIST counteracts activation or repression of IFN-γ-responsive promoters with different chromatin landscapes mechanistically.

## Materials and Methods

### Host Cells, Parasites and Infection

The murine leukemia monocyte/macrophage cell line RAW264.7 (TIB 71; ATCC, Rockville, MD, USA) was cultured in RPMI 1640 containing 4.5 g/l glucose, 10% FCS, 1 mM sodium pyruvate, 10 mM HEPES, 100 U/ml penicillin and 100 μg/ml streptomycin. Tachyzoites of the mouse-avirulent type II *T. gondii* strain NTE (62) were propagated in L929 fibroblasts as described previously (17). Prior to infection, parasites were isolated by differential centrifugation and thoroughly washed (39). Unless stated otherwise, host cells were infected at a parasite-to-host cell ratio of 6:1 for 24 h. Infected cells or non-infected controls were stimulated with 100 U/ml (all experiments except ChIP) or 300 U/ml (ChIP) of recombinant mouse IFN-γ (R&D Systems, Wiesbaden, Germany) starting at 3–23.5 h after infection as indicated. For some experiments, RAW264.7 cells were treated with 0.5 μM of 5-aza-2-deoxycytidine (AZA) for 7 days prior to infection with *T. gondii* and/or treatment with IFN-γ.

### Plasmids and Transfection

The plasmid pGL3-mCIITAp1.4(WT)-*luc* encoding luciferase under control of the full-length *cIIta* pIV (63) was kindly provided by J. Ernst, New York, and was used to generate a plasmid with a 5′-truncated version of the *cIIta* pIV encompassing nucleotides −403 to +83 [designated pGL3-mCIITAp1.4(−403/+83)-*luc*]. In order to generate an IFN-γ primary response reporter, an oligonucleotide with four adjacent GAS consensus sequences (5′-AGTTTCATATTACTCTAAATCAGTTTCATATTACTCTAAATCAGTTTCATATTACTCTAAATCAGTTTCATATTACTCTAAAT-3′; GASs underlined) was cloned (ATG Biosynthetics, Merzenhausen, Germany) into the pGL4.27[Luc2P/minP/Hygro] vector (Promega, Madison, WI, USA) and was referred to as pGL4.27-4 × GAS-*luc*. Sequences of reporter constructs were verified by sequencing (Microsynth SeqLab, Göttingen, Germany). RAW264.7 monocytic cells were transfected using the X-treme Gene HP DNA Transfection Reagent as recommended by the manufacturer (Roche, Mannheim, Germany). Briefly, 2 μg of pDNA were complexed with 6 μl of transfection reagent and were added to 1 × 10^6^ RAW264.7 seeded 24 h prior to transfection. After 8 h, cells were isolated, reseeded and 12 h later, experimentally treated as indicated. Stable RAW264.7/4xGAS-*luc* reporter cells were selected using 150 μg/ml hygromycin B and cloned by limiting dilution. A mutant RAW264.7 cell line stably expressing luciferase under control of promoter region −477 to +83 of the *cIIta* promoter IV was described previously (64).

### *In silico* Prediction of CpG Islands

Nucleotide sequences from −200 to +200 upstream and downstream of the transcriptional start site (TSS) of representative IFN-γ-regulated genes were retrieved from the DataBase of TSSs (65). CpG islands were predicted either separately within DNA regions −200 to +1 or +1 to +200 or within the complete DNA region −200 to +200 using the CpG Island Searcher online resource (66). CpG islands were identified as nucleotide sequences of at least 100 bp with an observed CpG/expected CpG ratio of >0.6 and a GC content of >50% (67).

### DNA Methylation Analysis

Methylation of cytosines within promoters of representative primary and secondary IFN-γ response genes was determined by methylation-sensitive melting curve analysis (MS-MCA) following bisulfite treatment of genomic DNA (50). To this end, DNA from *T. gondii*-infected and non-infected RAW264.7 cells either stimulated or not with IFN-γ was isolated using the QIAamp DNA Mini Kit (Qiagen, Hilden, Germany) following the manufacturer's instructions. Equal amounts of DNA (up to 500 ng/sample) were then bisulfite-converted using the EZ DNA Methylation-Lightning Kit as recommended by the manufacturer (Zymo Research Europe, Freiburg, Germany). Universal Methylated Mouse DNA Standard (Zymo Research) was bisulfite-converted in parallel and was used as positive control. Subsequently, promoter regions of *irf1* and *cIIta* were amplified from bisulfite-converted and input DNA by PCR in a LightCycler 1.5 (Roche, Manheim, Germany) using the LightCycler FastStart DNA Master^Plus^ SYBR Green I kit (Roche). Primers (Supplementary Table 2) were designed to allow for methylation-independent target amplification following previously recommended guidelines (68, 69). They were assessed by calculating oligonucleotide properties (http://biotools.nubic.northwestern.edu/OligoCalc.html) and by comparing melting curves (https://www.dna.utah.edu/umelt/umelt.html) of the expected amplicons assuming that all cytosines within CpG dinucleotides were either methylated or non-methylated. Melting curves of amplicons were recorded between 70 and 99°C without initial denaturation and reannealing (50).

### Luciferase Reporter Assay

Luciferase reporter activity was quantitated in transiently or stably transfected RAW264.7 mutants or RAW264.7 wild type cells using the Luciferase Assay System according to the manufacturer's instructions (Promega, Madison, WI, USA). Briefly, *T. gondii*-infected and non-infected cells treated or not with 100 U/ml IFN-γ were lysed (4 × 10^4^ cells/μl of lysis buffer) and soluble material harvested after centrifugation at 12,000 × *g* at 4°C. After addition of 20 μl of protein lysate to 100 μl of luciferase substrate, luminescence was measured using a Victor^3^ V multi-label microplate reader (Perkin Elmer, Rodgau, Germany).

### Chromatin Immunoprecipitation (ChIP)

Histone modifications were analyzed in infected and non-infected RAW264.7 cells during stimulation with IFN-γ for 0–18 h. To this end, DNA-protein complexes were cross-linked by incubating cells in 1% formaldehyde in PBS, pH 7.4 for 10 min. Reactivity was quenched by adding 125 mM glycine (final concentration) for 5 min. After having been washed twice with ice-cold PBS, 1 × 10^7^ cells/sample were isolated and lysed in 320 mM sucrose, 10 mM HEPES, pH 8.0, 5 mM CaCl_2_, 5 mM magnesium acetate, 0.1 mM EDTA, pH 8.0, 1 mM DTT, 0.1% Triton X-100 and protease inhibitor cocktail (Roche Diagnostics, Mannheim, Germany) at 4°C. After centrifugation at 1,000 × *g* for 5 min, pellets containing nuclei were washed twice in 140 mM NaCl, 50 mM Tris, pH 8.0, 20 mM EDTA, pH 8.0, 0.5% NP-40, 1% Triton X-100 and protease inhibitor cocktail, and they were then extracted in 300 μl/sample RIPA buffer [140 mM NaCl, 10 mM Tris, pH 8.0, 1 mM EDTA, pH 8.0, 0.1% sodium deoxycholate (NaDOC), 1% Triton X-100, 0.5% SDS and protease inhibitor cocktail] for 10 min at 4°C under constant rotation. Chromatin was sheared to fragment lengths of 200–1,000 bp using a Bioruptor Plus sonifier (Diagenode, Seraing, Belgium). After centrifugation at 18,000 × *g* for 5 min, supernatants were stored at −80°C. To test efficient shearing of chromatin, 50 μl of supernatant were treated with 20 μg each of proteinase K and RNase A for 3 h at 55°C and overnight at 65°C. DNA was then purified using a PCR Purification kit (Qiagen, Hilden, Germany) and resolved by agarose gel electrophoresis.

Prior to ChIP, unspecific binding sites of magnetic Dynabeads Protein A (Invitrogen, Carlsbad, U.S.A.) were blocked during 2 h at 4°C with 0.5% bovine serum albumin in IP buffer (140 mM NaCl, 50 mM Tris, pH 8.0, 20 mM EDTA, pH 8.0, 1% NP-40 and 0.5% NaDOC). After resuspending the beads in IP buffer, sheared chromatin (100 μl, 1:10 diluted in IP buffer supplemented with protease inhibitor cocktail) were precleared with 20 μl of blocked Protein A beads for 1 h at 4°C. One μg/sample of precleared chromatin was then incubated overnight at 4°C with 2 μg of rabbit anti-acetyl-histone H4, rabbit anti-acetyl-histone H3K9, mouse anti-trimethyl-histone H3K4, mouse anti-phospho-histone H3S10 (clone CMA312) or isotype control antibodies (all antibodies from Merck Millipore, Darmstadt, Germany). Immune complexes were collected with 15 μl/sample of blocked Protein A magnetic beads. They were washed twice in IP buffer supplemented with 0.1% SDS, trice in 0.5 M LiCl, 100 mM Tris, pH 8.0, 20 mM EDTA, pH 8.0, 1% NP-40 and 1% NaDOC, and once in IP buffer. Beads and 100 ng of sheared input chromatin were then consecutively incubated for 30 min at 37°C with 2 μg of RNase A and overnight at 65°C with 20 μg of Proteinase K. After isolation of DNA using a PCR purification kit (see above), distal promoter regions of *irf1, irf8, stat1, cIIta, gbp2*, or β*-actin* were amplified by quantitative LightCycler PCR (see above) using primers as specified in Supplementary Table 2. The IFN-γ-induced regulation of histone modifications was normalized to input DNA and was calculated as in the below equation (70).

$$\begin{array}{l} \text{Ratio }\left(\text{IFN}\gamma /\text{unstimulated}\right)=\frac{2^{\Delta \text{CP ChIP}\left(\text{unstimulated}-\text{IFN}\gamma -\text{treated}\right)}}{2^{\Delta \text{CP input}\left(\text{unstimulated}-\text{IFN}\gamma -\text{treated}\right)}} \end{array}$$

### Flow Cytometry

Surface expression of MHC class II molecules H2-A/E on RAW264.7 cells was quantified by FACS (fluorescence-activated cell sorting) as described before (39). Briefly, infected and non-infected monocytes/macrophages were isolated at ~42 h of infection and were washed twice. Unspecific binding sites of 500,000 cells per staining were blocked with 1 mg/ml normal mouse IgG, 1% bovine serum albumin (BSA), 0.1% NaN_3_ in PBS, pH 7.4 during 30 min at 4°C. Cells were then incubated with 2 μg/mL of rat monoclonal anti-H2-A/E (clone M5/114.15.2 ATCC, Rockville, MD) or with a rat IgG2b isotype control antibody (clone A95-1; BD Biosciences, Heidelberg, Germany) for 30 min at 4°C. After having been washed three times in 1% BSA, 0.1% NaN_3_ in PBS, pH 7.4, immune complexes were labeled with R-PE-conjugated donkey F(ab′)_2_ fragment anti-rat IgG for 30 min at 4°C. Cells were then washed, and they were fixed using 1% paraformaldehyde in PBS, pH 7.4. Ten thousand cells per sample were analyzed using a FACSCalibur (BD Biosciences).

### HDAC and HAT Activity Tests

HDAC and HAT activities were quantitated in nuclear extracts from infected and non-infected RAW264.7 cells stimulated with IFN-γ for 0–21 h. To this end, after collection, monocytic cells were incubated for 15 min at 4°C in hypotonic lysis buffer (10 mM HEPES, pH 7.8, 10 mM KCl, 2 mM MgCl_2_, 1 mM DTT, 0.1 mM EDTA, 0.1 mM PMSF, 0.1 mM Na_3_VO_4_). They were then disrupted by adding 0.6% Nonidet P-40, vigorous mixing and passage through a 26G needle. Complete cell lysis was assured microscopically after trypan blue staining. After centrifugation at 10,000 × *g* and 4°C for 1 min, the pellet was washed in hypotonic lysis buffer (as above), before being extracted in 50 mM HEPES, 50 mM KCl, 300 mM NaCl, 1 mM DTT, 0.1 mM EDTA, 0.1 mM PMSF, 0.1 mM Na_3_PO_4_ and 10% glycerol for 20 min at 4°C. Soluble nuclear proteins were collected after centrifugation at 14,000 × *g* for 5 min.

HDAC activity was determined using the Fluor de Lys®-Green fluorometric test kit as recommended by the manufacturer (Enzo Life Sciences, Lörrach, Germany). Briefly, 10 μl of nuclear extracts originating from ~2 × 10^4^ RAW264.7 cells each were incubated in duplicate with 200 μM of Fluor de Lys® substrate in HDAC assay buffer for 15 min at 37°C. In some experiments, 1 μM of HDAC inhibitor trichostatin A or 3 mM of NAD^+^ were added in parallel in order to confirm specificity of the test or to determine the impact of sirtuin-type HDACs on deacetylation, respectively. After addition of Fluor de Lys® developer containing 2 μM of trichostatin A, fluorescence was measured at excitation and emission wavelengths of 485 and 535 nm, respectively, in a Victor^3^ V multi-label microplate reader (Perkin Elmer).

HAT activity was quantitated using the HAT assay kit as recommended (Upstate, Lake Placid, NY). Briefly, 0.1 μg each of biotin-conjugated histone H3 or H4 peptides per well were incubated overnight at 4°C in streptavidin-coated microtitre plates. After blocking unspecific binding sites with 3% BSA in Tris-buffered saline (TBS) for 30 min at 30°C, 10 μl/well (histone H4; ~2 × 10^6^ RAW264.7 cells) or 20 μl/well (histone H3; ~4 × 10^6^ RAW264.7) of nuclear extracts, 100 μM acetyl-CoA, in 50 mM Tris, pH 8.0, 10% glycerol, 0.1 mM EDTA and 1 mM DTT were incubated in duplicate for 60 min at 30°C. Plates were then extensively washed, and acetylated peptides were consecutively labeled with 40 ng/well of rabbit IgG anti-acetyl-lysine and HRPO-conjugated anti-rabbit IgG. Bound antibodies were colorimetrically quantitated at 450 nm using a microplate reader.

### Statistical Analyses

Results are expressed as means ± S.E.M. of at last three independent experiments unless stated otherwise. Significant differences between means of two or more variables were identified by Student's *t*-test or by ANOVA with Bonferroni *post-hoc* test, respectively using Statistica 13 (Dell, Round Rock, USA). *P*-values of < 0.05 were considered significant.

## Data Availability Statement

All datasets generated for this study are included in the article/Supplementary Material.

## Author Contributions

RN and TC performed the experiments and analyzed data. CL conceived the study, analyzed data, and drafted the manuscript. All authors contributed to the article and approved the submitted version.

## Conflict of Interest

The authors declare that the research was conducted in the absence of any commercial or financial relationships that could be construed as a potential conflict of interest.

## References

[1] Montoya JG Liesenfeld O. Toxoplasmosis. Lancet. (2004) 363:1965–76. 10.1016/S0140-6736(04)16412-X 15194258

[2] Pleyer U Schluter D Manz M. Ocular toxoplasmosis: recent aspects of pathophysiology and clinical implications. Ophthalmic Res. (2014) 52:116–23. 10.1159/000363141 25248050

[3] Jones JL Muccioli C Belfort R Jr Holland GN Roberts JM Silveira C. Recently acquired *Toxoplasma gondii* infection, Brazil. Emerg Infect Dis. (2006) 12:582–7. 10.3201/eid1204.051081 16704805 PMC3294697

[4] Hoffmann S Batz MB Morris JG Jr. Annual cost of illness and quality-adjusted life year losses in the United States due to 14 foodborne pathogens. J Food Protoc. (2012) 75:1292–302. 10.4315/0362-028X.JFP-11-417 22980013

[5] Hunter CA Sibley LD. Modulation of innate immunity by *Toxoplasma gondii* virulence effectors. Nat Rev Microbiol. (2012) 10:766–78. 10.1038/nrmicro2858 23070557 PMC3689224

[6] Hakimi MA Olias P Sibley LD. Toxoplasma effectors targeting host signaling and transcription. Clin Microbiol Rev. (2017) 30:615–45. 10.1128/CMR.00005-17 28404792 PMC5475222

[7] Lambert H Hitziger N Dellacasa I Svensson M Barragan A. Induction of dendritic cell migration upon *Toxoplasma gondii* infection potentiates parasite dissemination. Cell Microbiol. (2006) 8:1611–23. 10.1111/j.1462-5822.2006.00735.x 16984416

[8] Courret N Darche S Sonigo P Milon G Buzoni-Gatel D Tardieux I CD11c- and CD11b-expressing mouse leukocytes transport single *Toxoplasma gondii* tachyzoites to the brain. Blood. (2006) 107:309–16. 10.1182/blood-2005-02-0666 16051744 PMC1895351

[9] Saeij JP Coller S Boyle JP Jerome ME White MW Boothroyd JC. Toxoplasma co-opts host gene expression by injection of a polymorphic kinase homologue. Nature. (2007) 445:324–7. 10.1038/nature05395 17183270 PMC2637441

[10] Taylor S Barragan A Su C Fux B Fentress SJ Tang K A secreted serine-threonine kinase determines virulence in the eukaryotic pathogen *Toxoplasma gondii*. Science. (2006) 314:1776–80. 10.1126/science.1133643 17170305

[11] Rosowski EE Lu D Julien L Rodda L Gaiser RA Jensen KD. Strain-specific activation of the NF-kappaB pathway by GRA15, a novel *Toxoplasma gondii* dense granule protein. J Exp Med. (2011) 208:195–212. 10.1084/jem.20100717 21199955 PMC3023140

[12] Bougdour A Durandau E Brenier-Pinchart MP Ortet P Barakat M Kieffer S. Host cell subversion by Toxoplasma GRA16, an exported dense granule protein that targets the host cell nucleus and alters gene expression. Cell Host Microbe. (2013) 13:489–500. 10.1016/j.chom.2013.03.002 23601110

[13] Braun L Brenier-Pinchart MP Yogavel M Curt-Varesano A Curt-Bertini RL Hussain T. A Toxoplasma dense granule protein, GRA24, modulates the early immune response to infection by promoting a direct and sustained host p38 MAPK activation. J Exp Med. (2013) 210:2071–86. 10.1084/jem.20130103 24043761 PMC3782045

[14] Hammoudi PM Jacot D Mueller C Di Cristina M Dogga SK Marq JB Fundamental roles of the golgi-associated toxoplasma aspartyl protease, ASP5, at the host-parasite interface. PLoS Pathog. (2015) 11:e1005211 10.1371/journal.ppat.1005211 26473595 PMC4608785

[15] Franco M Panas MW Marino ND Lee MC Buchholz KR Kelly FD. A novel secreted protein, MYR1, is central to toxoplasma's manipulation of host cells. MBio. (2016) 7:e02231–15. 10.1128/mBio.02231-15 26838724 PMC4742717

[16] Naor A Panas MW Marino N Coffey MJ Tonkin CJ Boothroyd JC. MYR1-dependent effectors are the major drivers of a host cell's early response to toxoplasma, including counteracting MYR1-independent effects. MBio. (2018) 9:e02401–17. 10.1128/mBio.02401-17 29615509 PMC5885026

[17] Lüder CG Lang T Beuerle B Gross U. Down-regulation of MHC class II molecules and inability to up-regulate class I molecules in murine macrophages after infection with *Toxoplasma gondii*. Clin Exp Immunol. (1998) 112:308–16. 10.1046/j.1365-2249.1998.00594.x 9649196 PMC1904980

[18] Lüder CG Algner M Lang C Bleicher N Gross U. Reduced expression of the inducible nitric oxide synthase after infection with *Toxoplasma gondii* facilitates parasite replication in activated murine macrophages. Int J Parasitol. (2003) 33:833–44. 10.1016/S0020-7519(03)00092-4 12865083

[19] Kim SK Fouts AE Boothroyd JC. *Toxoplasma gondii* dysregulates IFN-gamma-inducible gene expression in human fibroblasts: insights from a genome-wide transcriptional profiling. J Immunol. (2007) 178:5154–65. 10.4049/jimmunol.178.8.5154 17404298

[20] Lang C Hildebrandt A Brand F Opitz L Dihazi H Luder CG. Impaired chromatin remodelling at STAT1-regulated promoters leads to global unresponsiveness of *Toxoplasma gondii*-infected macrophages to IFN-gamma. PLoS Pathog. (2012) 8:e1002483. 10.1371/journal.ppat.1002483 22275866 PMC3262016

[21] Boehm U Klamp T Groot M Howard JC. Cellular responses to interferon-gamma. Annu Rev Immunol. (1997) 15:749–95. 10.1146/annurev.immunol.15.1.749 9143706

[22] Shuai K Schindler C Prezioso VR Darnell JE Jr. Activation of transcription by IFN-gamma: tyrosine phosphorylation of a 91-kD DNA binding protein. Science. (1992) 258:1808–12. 10.1126/science.1281555 1281555

[23] Suzuki Y Orellana MA Schreiber RD Remington JS. Interferon-gamma: the major mediator of resistance against *Toxoplasma gondii*. Science. (1988) 240:516–8. 10.1126/science.3128869 3128869

[24] Lieberman LA Banica M Reiner SL Hunter CA. STAT1 plays a critical role in the regulation of antimicrobial effector mechanisms, but not in the development of Th1-type responses during toxoplasmosis. J Immunol. (2004) 172:457–63. 10.4049/jimmunol.172.1.457 14688355

[25] Gavrilescu LC Butcher BA Del Rio L Taylor GA Denkers EY. STAT1 is essential for antimicrobial effector function but dispensable for gamma interferon production during *Toxoplasma gondii* infection. Infect Immun. (2004) 72:1257–64. 10.1128/IAI.72.3.1257-1264.2004 14977926 PMC356043

[26] Olias P Etheridge RD Zhang Y Holtzman MJ Sibley LD. Toxoplasma effector recruits the Mi-2/NuRD complex to repress STAT1 transcription and block IFN-gamma-dependent gene expression. Cell Host Microbe. (2016) 20:72–82. 10.1016/j.chom.2016.06.006 27414498 PMC4947229

[27] Gay G Braun L Brenier-Pinchart MP Vollaire J Josserand V Bertini RL. *Toxoplasma gondii* TgIST co-opts host chromatin repressors dampening STAT1-dependent gene regulation and IFN-gamma-mediated host defenses. J Exp Med. (2016) 213:1779–98. 10.1084/jem.20160340 27503074 PMC4995087

[28] Nast R Staab J Meyer T Luder CGK. *Toxoplasma gondii* stabilises tetrameric complexes of tyrosine-phosphorylated signal transducer and activator of transcription-1 and leads to its sustained and promiscuous DNA binding. Cell Microbiol. (2018) 20:e12887. 10.1111/cmi.12887 29968354

[29] Rosowski EE Nguyen QP Camejo A Spooner E Saeij JP. *Toxoplasma gondii* Inhibits gamma interferon (IFN-gamma)- and IFN-beta-induced host cell STAT1 transcriptional activity by increasing the association of STAT1 with DNA. Infect Immun. (2014) 82:706–19. 10.1128/IAI.01291-13 24478085 PMC3911376

[30] Medzhitov R Horng T. Transcriptional control of the inflammatory response. Nat Rev Immunol. (2009) 9:692–703. 10.1038/nri2634 19859064

[31] Bannister AJ Kouzarides T. Regulation of chromatin by histone modifications. Cell Res. (2011) 21:381–95. 10.1038/cr.2011.22 21321607 PMC3193420

[32] Deaton AM Bird A. CpG islands and the regulation of transcription. Genes Dev. (2011) 25:1010–22. 10.1101/gad.2037511 21576262 PMC3093116

[33] Antequera F. Structure, function and evolution of CpG island promoters. Cell Mol Life Sci. (2003) 60:1647–58. 10.1007/s00018-003-3088-6 14504655 PMC11138798

[34] Saxonov S Berg P Brutlag DL. A genome-wide analysis of CpG dinucleotides in the human genome distinguishes two distinct classes of promoters. Proc Natl Acad Sci USA. (2006) 103:1412–7. 10.1073/pnas.0510310103 16432200 PMC1345710

[35] Suzuki MM Bird A. DNA methylation landscapes: provocative insights from epigenomics. Nat Rev Genet. (2008) 9:465–76. 10.1038/nrg2341 18463664

[36] Zhang Y Ng HH Erdjument-Bromage H Tempst P Bird A Reinberg D. Analysis of the NuRD subunits reveals a histone deacetylase core complex and a connection with DNA methylation. Genes Dev. (1999) 13:1924–35. 10.1101/gad.13.15.1924 10444591 PMC316920

[37] Gao H Lukin K Ramirez J Fields S Lopez D Hagman J. Opposing effects of SWI/SNF and Mi-2/NuRD chromatin remodeling complexes on epigenetic reprogramming by EBF and Pax5. Proc Natl Acad Sci USA. (2009) 106:11258–63. 10.1073/pnas.0809485106 19549820 PMC2708696

[38] Choi WI Jeon BN Yoon JH Koh DI Kim MH Yu MY. The proto-oncoprotein FBI-1 interacts with MBD3 to recruit the Mi-2/NuRD-HDAC complex and BCoR and to silence p21WAF/CDKN1A by DNA methylation. Nucleic Acids Res. (2013) 41:6403–20. 10.1093/nar/gkt359 23658227 PMC3711425

[39] Lang C Algner M Beinert N Gross U Lüder CG. Diverse mechanisms employed by *Toxoplasma gondii* to inhibit IFN-gamma-induced major histocompatibility complex class II gene expression. Microbes Infect. (2006) 8:1994–2005. 10.1016/j.micinf.2006.02.031 16824778

[40] Ramirez-Carrozzi VR Braas D Bhatt DM Cheng CS Hong C Doty KR. A unifying model for the selective regulation of inducible transcription by CpG islands and nucleosome remodeling. Cell. (2009) 138:114–28. 10.1016/j.cell.2009.04.020 19596239 PMC2712736

[41] Meyer T Hendry L Begitt A John S Vinkemeier U. A single residue modulates tyrosine dephosphorylation, oligomerization, and nuclear accumulation of stat transcription factors. J Biol Chem. (2004) 279:18998–9007. 10.1074/jbc.M400766200 15010467

[42] Begitt A Droescher M Meyer T Schmid CD Baker M Antunes F. STAT1-cooperative DNA binding distinguishes type 1 from type 2 interferon signaling. Nat Immunol. (2014) 15:168–76. 10.1038/ni.2794 24413774

[43] Smale ST Natoli G. Transcriptional control of inflammatory responses. Cold Spring Harb Perspect Biol. (2014) 6:a016261. 10.1101/cshperspect.a016261 25213094 PMC4413233

[44] Bhatt DM Pandya-Jones A Tong AJ Barozzi I Lissner MM Natoli G. Transcript dynamics of proinflammatory genes revealed by sequence analysis of subcellular RNA fractions. Cell. (2012) 150:279–90. 10.1016/j.cell.2012.05.043 22817891 PMC3405548

[45] Kang K Park SH Chen J Qiao Y Giannopoulou E Berg K. Interferon-gamma represses M2 gene expression in human macrophages by disassembling enhancers bound by the transcription factor MAF. Immunity. (2017) 47:235–50 e4. 10.1016/j.immuni.2017.07.017 28813657 PMC5568089

[46] Ramirez J Dege C Kutateladze TG Hagman J. MBD2 and multiple domains of CHD4 are required for transcriptional repression by Mi-2/NuRD complexes. Mol Cell Biol. (2012) 32:5078–88. 10.1128/MCB.00819-12 23071088 PMC3510529

[47] Morris AC Spangler WE Boss JM. Methylation of class II trans-activator promoter IV: a novel mechanism of MHC class II gene control. J Immunol. (2000) 164:4143–9. 10.4049/jimmunol.164.8.4143 10754309

[48] Morris AC Beresford GW Mooney MR Boss JM. Kinetics of a gamma interferon response: expression and assembly of CIITA promoter IV and inhibition by methylation. Mol Cell Biol. (2002) 22:4781–91. 10.1128/MCB.22.13.4781-4791.2002 12052885 PMC133907

[49] Satoh A Toyota M Ikeda H Morimoto Y Akino K Mita H. Epigenetic inactivation of class II transactivator (CIITA) is associated with the absence of interferon-gamma-induced HLA-DR expression in colorectal and gastric cancer cells. Oncogene. (2004) 23:8876–86. 10.1038/sj.onc.1208144 15467734

[50] Worm J Aggerholm A Guldberg P. In-tube DNA methylation profiling by fluorescence melting curve analysis. Clin Chem. (2001) 47:1183–9. 10.1093/clinchem/47.7.1183 11427447

[51] Wang Z Schones DE Zhao K Characterization of human epigenomes. Curr Opin Genet Dev. (2009) 19:127–34. 10.1016/j.gde.2009.02.001 19299119 PMC2699568

[52] Schneider AG Abi Abdallah DS Butcher BA Denkers EY. *Toxoplasma gondii* triggers phosphorylation and nuclear translocation of dendritic cell STAT1 while simultaneously blocking IFNgamma-induced STAT1 transcriptional activity. PLoS ONE. (2013) 8:e60215. 10.1371/journal.pone.0060215 23527309 PMC3603897

[53] Vaughan EE DeGiulio JV Dean DA. Intracellular trafficking of plasmids for gene therapy: mechanisms of cytoplasmic movement and nuclear import. Curr Gene Ther. (2006) 6:671–81. 10.2174/156652306779010688 17168698 PMC4400175

[54] Pattenden SG Klose R Karaskov E Bremner R. Interferon-gamma-induced chromatin remodeling at the CIITA locus is BRG1 dependent. EMBO J. (2002) 21:1978–86. 10.1093/emboj/21.8.1978 11953317 PMC125964

[55] Ramirez-Carrozzi VR Nazarian AA Li CC Gore SL Sridharan R Imbalzano AN. Selective and antagonistic functions of SWI/SNF and Mi-2beta nucleosome remodeling complexes during an inflammatory response. Genes Dev. (2006) 20:282–96. 10.1101/gad.1383206 16452502 PMC1361700

[56] Hargreaves DC Horng T Medzhitov R. Control of inducible gene expression by signal-dependent transcriptional elongation. Cell. (2009) 138:129–45. 10.1016/j.cell.2009.05.047 19596240 PMC2828818

[57] Glass CK Natoli G. Molecular control of activation and priming in macrophages. Nat Immunol. (2016) 17:26–33. 10.1038/ni.3306 26681459 PMC4795476

[58] Qiao Y Giannopoulou EG Chan CH Park SH Gong S Chen J. Synergistic activation of inflammatory cytokine genes by interferon-gamma-induced chromatin remodeling and toll-like receptor signaling. Immunity. (2013) 39:454–69. 10.1016/j.immuni.2013.08.009 24012417 PMC3857147

[59] Wang Z Zang C Rosenfeld JA Schones DE Barski A Cuddapah S. Combinatorial patterns of histone acetylations and methylations in the human genome. Nat Genet. (2008) 40:897–903. 10.1038/ng.154 18552846 PMC2769248

[60] Davie JR. MSK1 and MSK2 mediate mitogen- and stress-induced phosphorylation of histone H3: a controversy resolved. Sci STKE. (2003) 2003:PE33. 10.1126/stke.2003.195.pe33 12915720

[61] Leng J Butcher BA Egan CE Abdallah DS Denkers EY. *Toxoplasma gondii* prevents chromatin remodeling initiated by TLR-triggered macrophage activation. J Immunol. (2009) 182:489–97. 10.4049/jimmunol.182.1.489 19109180 PMC2651083

[62] Gross U Muller WA Knapp S Heesemann J. Identification of a virulence-associated antigen of *Toxoplasma gondii* by use of a mouse monoclonal antibody. Infect Immun. (1991) 59:4511–6. 10.1128/IAI.59.12.4511-4516.1991 1718876 PMC259071

[63] O'Keefe GM Nguyen VT Ping Tang LL Benveniste EN. IFN-gamma regulation of class II transactivator promoter IV in macrophages and microglia: involvement of the suppressors of cytokine signaling-1 protein. J Immunol. (2001) 166:2260–9. 10.4049/jimmunol.166.4.2260 11160280

[64] Kincaid EZ Ernst JD. Mycobacterium tuberculosis exerts gene-selective inhibition of transcriptional responses to IFN-gamma without inhibiting STAT1 function. J Immunol. (2003) 171:2042–9. 10.4049/jimmunol.171.4.2042 12902509

[65] Yamashita R Sugano S Suzuki Y Nakai K. DBTSS: database of transcriptional start sites progress report in 2012. Nucleic Acids Res. (2012) 40(Database issue):D150–4. 10.1093/nar/gkr1005 22086958 PMC3245115

[66] Takai D Jones PA. The CpG island searcher: a new WWW resource. In Silico Biol. (2003) 3:235–40. 12954087

[67] Gardiner-Garden M Frommer M. CpG islands in vertebrate genomes. J Mol Biol. (1987) 196:261–82. 10.1016/0022-2836(87)90689-9 3656447

[68] Clark SJ Harrison J Paul CL Frommer M. High sensitivity mapping of methylated cytosines. Nucleic Acids Res. (1994) 22:2990–7. 10.1093/nar/22.15.2990 8065911 PMC310266

[69] Wojdacz TK Hansen LL Dobrovic A. A new approach to primer design for the control of PCR bias in methylation studies. BMC Res Notes. (2008) 1:54. 10.1186/1756-0500-1-54 18710507 PMC2525644

[70] Pfaffl MW. A new mathematical model for relative quantification in real-time RT-PCR. Nucleic Acids Res. (2001) 29:e45. 10.1093/nar/29.9.e45 11328886 PMC55695

