# Supplementary material for: Epigenetic Control of IFN-γ Host Responses During Infection With Toxoplasma gondii
Source: Front Immunol. 2020 Sep 25;11:581241. doi: 10.3389/fimmu.2020.581241 (PMC7544956; doi:10.3389/fimmu.2020.581241)
Supplement: Supplementary file 1 [file Data_Sheet_1.pdf]

Supplementary Material

1 Supplementary Figures and Tables

1.1 Supplementary Figures

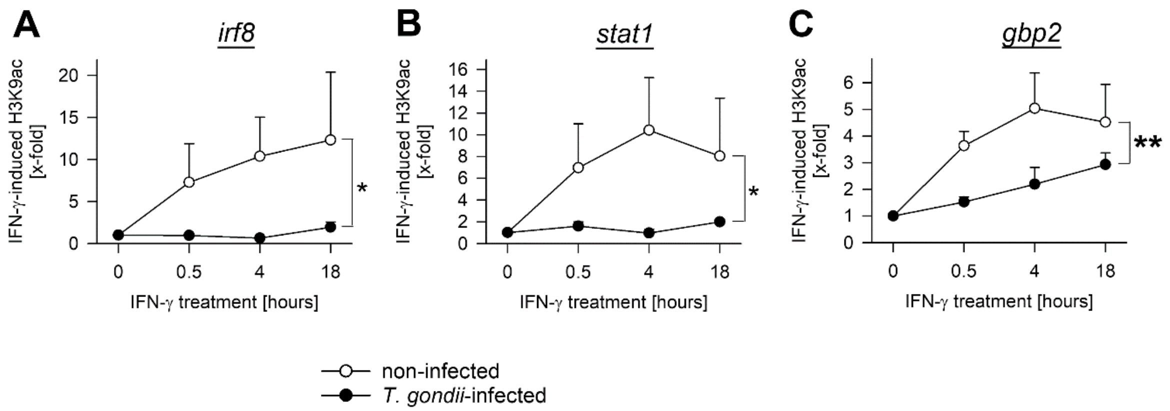

Supplementary Fig. S1. *T. gondii* inhibits acetylation of lysine 9 of histone H3 at promoters of primary and secondary response genes during IFN-γ stimulation. RAW264.7 cells were parasite-infected for 24 hours or were left non-infected. During the final 0.5 to 18 hours they were stimulated with IFN-γ or were left unstimulated (0 hours). After cross-linking DNA-protein complexes, cell lysates were subjected to ChIP using antibodies specific for acetylK9-H3. After isolation of DNA from chromatin immunoprecipitates or from input chromatin, fragments of promoters of *irf8* (**A**), *stat1* (**B**) and *gbp2* (**C**) were amplified by qPCR. Data indicates the means ± S.E.M. ($n \geq 2$) of cytokine-induced acetylation in *T. gondii*-infected (closed symbols) and non-infected cells (open symbols) normalized to input DNA; differences between experimental groups were identified by ANOVA (*$p < 0.05$; **$p < 0.01$).

1.2 Supplementary Tables

Supplementary Table S1. *In silico* identification of CpG islands in the promoters of IFN-γ-responsive genes regulated or not by *T. gondii*.

			IFN-γ / Ctrl. [x-fold]			CpG / Expected[a]		% GC[b]	
**Class**	**Gene ID**	**RefSeq**	**n.i.**	***T. gondii***	**Effect of *T. gondii* [x-fold]**	**-200/+1**	**+1/+200**	**-200/+1**	**+1/+200**
	Ptgs2[c]	NM_011198	59,39	0,28	0,00	0,64		53,20	
	Gbp4	NM_008620	4544,27	37,28	0,01				
	Timd4	NM_178759	239,15	2,66	0,01				
	Gbp1	NM_010259	1690,03	45,23	0,03				
	Iigp1	NM_021792	427,49	13,56	0,03				
	C1s	NM_001097617	30,93	1,01	0,03				
	Itgal	NM_008400	37,47	1,33	0,04				
	Igtp	NM_018738	88,57	3,65	0,04				
	Scd2	NM_009128	10,48	0,44	0,04		0,78		51,20
	Clec9a	NM_172732	1040,27	44,15	0,04				
IFN-γ-induced, *T. gondii*-inhibited	Dna2	NM_177372	6,01	0,27	0,04	1,13	0,98	72,10	72,10
	Wars	NM_011710	45,59	2,07	0,05				
	Mpa2l	NM_194336	237,48	10,84	0,05				
	H2-DMa	NM_010386	37,02	1,78	0,05				
	Ly6f	NM_008530	400,26	20,97	0,05				
	Gbp3	NM_018734	153,46	9,11	0,06				
	Smpdl3b	NM_133888	36,62	2,18	0,06	0,62	0,66	51,20	66,20
	Rnase6	NM_030098	71,34	4,50	0,06				
	Itpka	NM_146125	33,47	2,44	0,07	0,89	0,93	75,10	80,60
	Trpm2	NM_138301	10,32	0,77	0,07				
	Fam26f	NM_175449	145,40	10,87	0,07				
	Pstpip2	NM_013831	11,87	0,89	0,08	0,91	0,84	73,60	68,30

Stat1	NM_009283	19,98	1,55	0,08	0,83	0,75	64,20	63,20
Tgtp	NM_011579	162,83	13,04	0,08				
Tnni3	NM_009406	4,94	0,40	0,08				
Irf1	NM_008390	50,14	4,38	0,09	1,13	1,24	65,20	64,20
Ppargc1b	NM_133249	10,84	0,95	0,09	0,82	0,90	74,60	69,70
Dnase1l3	NM_007870	158,26	14,24	0,09				
Klhl6	NM_183390	5,17	0,47	0,09				
Gbp2	NM_010260	443,01	41,18	0,09				
Klf2	NM_008452	4,12	0,39	0,09	0,89	0,65	70,10	65,70
Pim1	NM_008842	7,67	0,73	0,10	0,83	0,85	75,10	73,60
Ciita	NM_007575	32,04	3,17	0,10				
Slamf8	NM_029084	19,26	2,07	0,11				
Klra2	NM_008462	17,90	1,94	0,11				
Ly6a	NM_010738	282,94	31,17	0,11				
Il4ra	NM_001008700	6,27	0,71	0,11	0,90	0,75	62,20	70,10
Pion	NM_175437	5,07	0,59	0,12		0,70		64,70
Ccl8	NM_021443	51,07	6,08	0,12				
Myh10	NM_175260	11,57	1,40	0,12	0,63	0,81	61,70	66,20
Irgm2	NM_019440	16,56	2,01	0,12				
Klrk1	NM_001083322	152,24	18,52	0,12				
Tap1	NM_013683	20,71	2,57	0,12	1,04		61,20	
Txn1	NM_011660	7,87	1,00	0,13		1,06		68,70
Saa3	NM_011315	56,83	7,20	0,13				
Fbxl5	NM_178729	4,96	0,63	0,13	1,15	0,97	82,10	73,60
Mmp25	NM_001033339	50,79	6,62	0,13	0,70		70,10	
H2-Ab1	NM_207105	74,48	9,70	0,13				
Irg1	NM_008392	99,73	13,02	0,13				
Ly6i	NM_020498	344,80	45,27	0,13				
Tnfrsf14	NM_178931	7,72	1,01	0,13				
Serpina3f	NM_001033335	2286,61	304,25	0,13				
Upp1	NM_009477	5,46	0,73	0,13				

	Ankrd33	NM_144790	5,06	0,68	0,13				
	H2-Eb1	NM_010382	42,09	5,69	0,14				
	Etv2	NM_007959	4,75	0,66	0,14				
	Apol7c	NM_175391	9,99	1,41	0,14				
	Ifi47	NM_008330	27,60	3,95	0,14				
	Gpd2	NM_010274	6,11	0,88	0,14	0,63	1,02	67,20	76,60
	H2-DMb1	NM_010387	110,22	15,92	0,14				
	Pnpla5	NM_029427	12,24	1,79	0,15				
	H2-Aa	NM_010378	72,93	10,68	0,15				
	Pdcd1lg2	NM_021396	8,61	1,27	0,15				
	Pla2g4a	NM_008869	21,56	3,22	0,15				
	Serpinb9	NM_009256	7,67	1,17	0,15				
	Bst1	NM_009763	24,20	3,74	0,15				
	Obfc2a	NM_028696	4,30	0,66	0,15	0,91	0,97	67,70	66,70

IFN-γ-induced, not inhibited by *T. gondii*	Glipr2	NM_027450	4,41	3,08	0,70	0,65	0,69	70,6	68,2
	Il18bp	NM_010531	18,47	12,88	0,70				
	Hoxb5	NM_008268	4,59	3,20	0,70				
	Abtb2	NM_178890	4,63	3,24	0,70	0,80	1,03	61,2	63,2
	Ap1s3	NM_183027	4,17	2,92	0,70		0,64		66,2
	D14Ertd668e	NM_199015	9,80	6,87	0,70				
	Fam82a2	NM_001033136	4,58	3,24	0,71	1,07	0,74	64,2	69,2
	Usp12	NM_011669	5,10	3,67	0,72	0,86	1,02	79,1	84,1
	Cst7	NM_009977	15,09	10,97	0,73				
	Aif1	NM_019467	9,44	6,92	0,73				
	Lair1	NM_001113474	9,50	7,01	0,74				
	H28	NM_031367	4,38	3,24	0,74	0,61*		51,8*	
	Tapbp	NM_001025313	7,89	5,86	0,74	0,61		53,3	
	Chst4	NM_011998	4,09	3,05	0,75				
	Olfr703	NM_146596	4,38	3,28	0,75				

Cd74	NM_001042605	11,26	8,46	0,75				
Gtpbp2	NM_019581	6,75	5,08	0,75	0,77	0,86	58,2	74,1
Slc19a3	NM_030556	4,32	3,28	0,76	0,61*		53,6*	
Gls	NM_001081081	7,93	6,11	0,77	0,70	0,99	63,2	74,1
Ass1	NM_007494	8,17	6,33	0,77	0,67		79,1	
Psmb10	NM_013640	4,94	3,83	0,78				
Fbxo36	NM_025386	5,10	4,04	0,79		0,63		61,7
Zc3h12d	NM_172785	4,49	3,58	0,80				
Il17c	NM_145834	4,03	3,25	0,81				
Tmsb10	NM_001039392	6,09	4,92	0,81	0,92	0,72	65,2	66,7
Parp9	NM_030253	5,54	4,51	0,81		0,71		60,2
Mthfd2	NM_008638	4,53	3,70	0,82		1,01		70,1
Wdyhv1	NM_029734	4,26	3,56	0,84	0,70	1,05	70,1	67,7
C130026I21Rik	NM_001037909	4,21	3,54	0,84				
Psme1	NM_011189	4,41	3,71	0,84	0,66		69,7	
2310046K01Rik	NM_027172	4,30	3,68	0,85	0,80		61,2	
Padi6	NM_153106	7,00	6,14	0,88				
9030625A04Rik	NM_172488	7,25	6,38	0,88	0,94	1,02	79,6	76,1
Il18bp	NM_010531	12,30	10,89	0,89				
Slc2a6	NM_172659	4,11	3,71	0,90	0,83	0,63	67,7	61,7
Gnb2l1	NM_008143	6,00	5,54	0,92	0,76	0,72	54,2	60,2
Frem2	NM_172862	4,75	4,47	0,94		0,65		65,7
Pnp1	NM_013632	6,31	5,95	0,94		0,82		63,2
Mlkl	NM_029005	4,85	4,63	0,95	0,93		64,7	
S100a14	NM_025393	6,33	6,04	0,95				
Sla2	NM_029983	5,36	5,19	0,97				
Sdc3	NM_011520	4,34	4,21	0,97	0,97	1,26	79,6	79,6
4932415M13Rik	NM_001037718	12,03	11,86	0,99				
Gabrb3	NM_001038701	4,16	4,18	1,00	0,71		56,7	
Tgfbi	NM_009369	4,28	4,35	1,01	0,66	0,70	67,2	68,2
Gjd3	NM_178596	6,96	7,18	1,03		0,86		63,7

	Inpp5b	NM_008385	5,34	5,54	1,04	0,65		55,2	
	Tyk2	NM_018793	4,53	4,92	1,09	1,10		55,2	
	Parp11	NM_181402	4,05	4,42	1,09	1,06	0,81	64,7	70,1
	Casp1	NM_009807	4,34	4,75	1,09				
	P2ry14	NM_001008497	6,79	7,69	1,13				
	Phf11	NM_172603	6,12	7,23	1,18				
	Usp37	NM_176972	4,19	5,03	1,20				
	Gnb4	NM_013531	5,36	6,62	1,23	1,14	1,07	82,6	77,6
	Ptprz1	NM_001081306	8,62	10,99	1,27	0,80	0,89	75,1	63,7
	Tgs1	NM_054089	4,42	5,66	1,28	0,97	1,10	64,2	61,7
	Epas1	NM_010137	4,03	5,16	1,28	1,13		52,2	
	Clcn7	NM_011930	4,47	5,80	1,30	0,75	0,91	55,7	62,2
	Trafd1	NM_172275	4,96	6,48	1,31	0,67	1,06	67	83,6
	Arid5a	NM_145996	4,60	6,01	1,31	0,77	1,02	63,2	68,7
	Fam161a	NM_028672	4,26	5,74	1,35				
	Slfn1	NM_011407	8,19	11,18	1,36				
	Gpr114	NM_001033468	9,66	13,95	1,44				
	H2-T22	NM_010397	6,74	9,99	1,48				
	Cd69	NM_001033122	19,58	29,68	1,52				
	Tpst1	NM_001130476	4,22	6,61	1,57				
	Nos2	NM_010927	7,00	11,23	1,60				

	Mmp12	NM_008605	0,00	0,24	122,71				
	Strn	NM_011500	0,05	5,31	109,41	0,81	0,85	76,6	75,6
	Cyp4f39	NM_177307	0,06	6,17	98,39		0,61		59,7
IFN-γ-repressed, *T. gondii*-inhibited	Fabp12	NM_029310	0,05	4,96	96,09	0,66		52,2	
	Ccl4	NM_013652	0,19	17,77	94,32				
	Pcp4l1	NM_025557	0,05	3,98	85,37		0,68		66,7
	Fabp4	NM_024406	0,01	0,55	66,72				
	Cd28	NM_007642	0,02	0,95	62,55				

Lhfpl2	NM_172589	0,01	0,75	55,82	0,88	0,82	82,1	79,1
Ccl3	NM_011337	0,08	4,64	55,48				
Spp1	NM_009263	0,02	1,01	42,54				
Serpine1	NM_008871	0,07	2,92	39,02				
Ms4a7	NM_001025610	0,03	1,06	35,91				
Slc24a3	NM_053195	0,24	7,67	31,85				
Wdtc1	NM_199306	0,13	4,07	31,14	1,04	0,84	72,1	71,1
Emp1	NM_010128	0,01	0,36	30,84				
Cxcr4	NM_009911	0,01	0,33	30,70	0,92		60,7	
Bmp2	NM_007553	0,08	2,36	30,39	0,63	0,61	66,2	60,5
2310030G06Rik	NM_025865	0,18	4,23	23,24	0,61*		58,6*	
Ctsl	NM_009984	0,07	1,46	21,69	0,74	0,71	69,7	60,7
Gpnmb	NM_053110	0,04	0,81	21,32	0,62*		52,9*	
Tfrc	NM_011638	0,03	0,53	21,06	0,95	0,89	68,2	73,1
Il7r	NM_008372	0,05	1,07	20,53				
4930486L24Rik	NM_178098	0,07	1,51	20,12				
Aadacl1	NM_178772	0,19	3,67	19,85	0,65	0,88	65,7	64,2
Dnmt3a	NM_007872	0,17	3,32	19,52	0,92	1,05	84,1	82,6
Gdf3	NM_008108	0,09	1,60	18,32				
Arsb	NM_009712	0,11	1,81	17,19	0,93		62,2	
Tlr7	NM_133211	0,17	2,88	17,00				
Chst7	NM_021715	0,11	1,65	15,59		0,75		63,7
Cnrip1	NM_029861	0,06	0,88	15,43				
Npy	NM_023456	0,09	1,36	15,17	0,73	0,62	72,1	65,2
Tanc1	NM_198294	0,15	2,15	14,69	0,65	0,84	77,1	77,1
Dusp4	NM_176933	0,05	0,71	14,46	0,72	0,73	62,7	61,7
Fabp5	NM_010634	0,10	1,43	14,41	0,78		70,1	
Id2	NM_010496	0,10	1,45	14,26	0,83	0,73	62,2	60,2
Tmod1	NM_021883	0,05	0,76	14,06	0,63	1,05	69,5	74,1
Il1rn	NM_001039701	0,17	2,40	14,02				
A930005I04Rik	NM_207277	0,14	1,88	13,13				

	Adfp	NM_007408	0,08	1,09	12,92	0,63*		61,1*	
	Abcb4	NM_008830	0,07	0,85	12,12	0,82	0,94	65,2	77,6
	Ccl9	NM_011338	0,08	1,01	12,05				
	Cpeb4	NM_026252	0,22	2,64	11,98				
	Slc40a1	NM_016917	0,01	0,12	11,51	0,75	0,85	69,2	59,7

IFN-γ-repressed, not inhibited by *T. gondii*	Cxcl1	NM_008176	0,11	0,20	1,86				
	Gmpr	NM_025508	0,23	0,41	1,81		1,02		72,6
	Itga9	NM_001113514	0,16	0,29	1,81				
	Gabarapl1	NM_020590	0,23	0,40	1,76		0,87		57,2
	Tom1l1	NM_028011	0,25	0,43	1,74	0,77	0,63	73,6	60,2
	Aplp2	NM_001102455	0,18	0,31	1,74	0,98	0,82	63,7	69,7
	Wbp5	NM_011712	0,18	0,32	1,74	0,61*		51,9*	
	Zranb3	NM_027678	0,24	0,41	1,73				
	Clec4n	NM_020001	0,21	0,37	1,70				
	Abca1	NM_013454	0,11	0,19	1,68	1,03	0,75	59,7	65,2
	Ear1	NM_007894	0,08	0,13	1,67				
	Gab1	NM_021356	0,17	0,28	1,66	0,82	0,66	71,6	74,1
	Aqp9	NM_022026	0,21	0,35	1,66				
	Rnf149	NM_001033135	0,24	0,39	1,64	0,79	1,07	71,1	77,1
	Osbpl11	NM_176840	0,23	0,37	1,64	0,80	0,72	58,7	71,1
	Gna12	NM_010302	0,20	0,31	1,60	0,95	0,99	65,2	84,1
	Stmn1	NM_019641	0,25	0,39	1,59	0,70	0,72	65,2	70,6
	Dab2	NM_001008702	0,24	0,38	1,59				
	Rapgef5	NM_175930	0,24	0,38	1,55				
	Glul	NM_008131	0,08	0,13	1,51	0,64	0,71	64,2	63,2
	Nt5dc2	NM_027289	0,22	0,32	1,50				
	Fam63a	NM_133858	0,20	0,30	1,48		0,66		62,7
	Rgs18	NM_022881	0,24	0,33	1,40				
	1110002B05Rik	NM_134054	0,22	0,31	1,40	0,96	0,74	70,1	68,7

	Trf	NM_133977	0,11	0,16	1,39	0,61*		61,6*	
	Arhgap18	NM_176837	0,21	0,29	1,35				
	Clec4b1	NM_027218	0,19	0,26	1,35				
	Cd163	NM_053094	0,20	0,28	1,35				
	Cd9	NM_007657	0,18	0,25	1,35	0,62	0,69	62,5	53,7
	Arhgap6	NM_009707	0,19	0,25	1,32		0,64		62,5
	Ncoa4	NM_001033988	0,20	0,27	1,30	0,80	0,80	54,7	65,2
	Pde2a	NM_001008548	0,13	0,16	1,26				
	Cx3cr1	NM_009987	0,14	0,17	1,25				
	Trem2	NM_031254	0,22	0,26	1,21				
	Nme4	NM_019731	0,23	0,27	1,20		0,98		70,1
	Cav2	NM_016900	0,22	0,25	1,16	0,73	0,70	78,6	56,2
	Man2a2	NM_172903	0,22	0,25	1,14	0,97	0,81	82,1	76,1
	Zdhhc14	NM_146073	0,19	0,22	1,14	0,74	0,97	75,6	72,1
	Itsn1	NM_001110275	0,24	0,27	1,12	0,88	1,13	63,7	74,6
	Itgb5	NM_010580	0,25	0,27	1,09	0,84	1,05	70,6	81,6
	Idh1	NM_001111320	0,12	0,13	1,07	0,72	0,82	58,7	51,7
	Wwp1	NM_177327	0,19	0,20	1,02	0,95	1,07	77,6	77,1
	Sesn1	NM_001013370	0,25	0,20	0,80	0,86	1,00	74,6	72,1
	Cxcl2	NM_009140	0,10	0,08	0,80				
	9030425E11Rik	NM_133733	0,21	0,14	0,70		0,72		62,7

[a]Ratios of observed CpGs / expected CpGs of > 0.6 within at least 100 bp were considered indicative for a CpG island.

[b]GC contents of > 50% within at least 100 bp were considered indicative for a CpG island.

[c]Genes that contain CpG islands within -200 to +1 or +1 to +200 or both are highlighted.

*Genes that contain CpG islands within -200 to +200 bp relative to their transcriptional start site.

Supplementary Table S2. Primer sequences used in this study.

Promoter	Accession No.	Forward primer	Reverse primer
DNA methylation analysis			
Irf1	M21066.1$_{(1-329)}$ + M21065.1	5’-GGGGAATTTCGTTAAGTGTTTAGATTTTTT-3’	5’-CGAAATACTAAAATCCTCTAACCACCT-3’
CIIta	AF000008.1$_{(1-372)}$ + AF100710.1	5’-CGTGGTGGATATTATTTTTTAGGGGAG-3’	5’-CCGTACTTCTAAATACTACCTACATACAAT-3’
ChIP analysis			
Irf1	AL596182.29	5'-GCTGAAGTAGGTGTCATAGAA-3'	5'-GGAACACCTTGTCCTTATG-3'
CIIta	AC122352.4	5'-CACAGTGGGCTCAGGGGAATATC-3'	5'-GACCTCGGATGACAGTGGTTACT-3'
β-actin	AC144818.4	5'-ATCAGGCAGTTCTCCAAAAGT-3'	5'-TTGCAAGAAGATGCCTCCAGATA-3'
Irf8	AC114819.4	5'-GAAACCTAGCCGTATACTTCTTGTA-3'	5'-CTCTCACCCGTCTGGCTTTTA-3'
Stat1	AC123752.8	5'-ACGGGAGAAGGGTAGATAAG-3'	5'-TACACAATTGACACCTCCATTAC-3'
Gbp2	AF109169.1	5'-AGATGTCAGTCTCAGTTTTG-3'	5'-CCAGTCATTTGTAGTATGTT-3'
